# Supplementary material for: Genome mining of cyclodipeptide synthases unravels unusual tRNA-dependent diketopiperazine-terpene biosynthetic machinery
Source: Nat Commun. 2018 Oct 5;9:4091. doi: 10.1038/s41467-018-06411-x (PMC6173783; doi:10.1038/s41467-018-06411-x)
Supplement: Supplementary file 1 — Supporting Information [file 41467_2018_6411_MOESM1_ESM.pdf]

Supporting Information

Genome mining of cyclodipeptide synthases unravels unusual tRNA-dependent diketopiperazine-terpene biosynthetic machinery

Yao et al

Supplementary Table 1. The primer pairs used for DmtB1-3 and DmtC1 protein expression[a]

Name	Sequence (5'-3')
DmtB1-FP	GGAATTCCATATGACGAACACCAACCCTTCG
DmtB1-RP	CCGCTCGAGTGACACGGGCCGGGTGACGACG
DmtB2-FP	GGAATTCCATATGACCAGCAGGACCGAAACC
DmtB2-RP	CCGCTCGAGTCACGGAAGCAGCCGGGG
DmtB3-FP	GGAATTCCATATGACAGCGCCCGCACCGC
DmtB3-RP	CCGCTCGAGTCATGCCGGGTCCCCGCCG
DmtC1-FP	CCGGAATTCACCCGCCAGGAAATGGACG
DmtC1-RP	CCGCTCGAGCGTCGAGTCGGCGGTCAGGG

[a]Underlined letters represent restriction sites.

Supplementary Table 2. The primer pairs used for heterologous expression in *Streptomyces coelicolor* M1146[a]

Name	Sequence (5'-3')
dmtB1C1-FP	GTGACGAACACCAACCCTTC
dmtA1B1C1-FP	ATGGTAGACGGCATCCGTATG
dmtC1-RP	GCTCTAGACGGGTTACGGACTGCGGGTTA
dmtB2C2-FP	ATGTCACAGCAGATGACACCG
dmtA2B2C2-FP	ATGCCGTACGTCTCTTCCTTC
dmtC2-RP	CTAGTCTAGATCAGGCGGCGGTGGCCGAC
dmtB3C3-FP	ATGACAGCGCCCGCACCGC
dmtA3B2C3-FP	ATGACCGTGATCGCCGCCTC
dmtC3-RP	CTAGTCTAGATCACCGGAGCGGGCTGAACG
pGFP	CCAATGCATCCGTCGCGGAAAGCTGGC
pGRP	GAACCGATCTCCTCGTTGGTG

[a]Underlined letters represent restriction sites. The primer pairs of dmtB1C1-FP/ dmtC1-RP and dmtA1B1C1-FP/ dmtC1-RP were used for *dmtB1C1* and *dmtA1B1C1* cloning. The primer pairs of dmtB2C2-FR/ dmtC2-FP and dmtA2B2C2-FP/ dmtC2-RP were used for *dmtB2C2* and *dmtA2B2C2* cloning. The primer pairs of dmtB3C3-FP/ dmtC3-RP and dmtA3B3C3-FP/ dmtC3-RP were used for *dmtB3C3* and *dmtA3B3C3* cloning. The 3'-OH of pGRP was phosphorylated. pGFP/RP was used for amplification of promotor P_{gapdh}.

Supplementary Table 3. The primer pairs used for DmtB1 and DmtB2 site-directed mutagenesis[a]

Name	Sequence (5'-3')
DmtB1 E206A-FP	ACCAGCACCGAGTGGCCTTCATCGACCGCATC
DmtB1 E206A-RP	GATGCGGTCGATGAAGGCCACTCGGTGCTGGT
DmtB1 E206P-FP	ACCAGCACCGAGTGCCGTTCATCGACCGCATC
DmtB1 E206P-RP	GATGCGGTCGATGAACGGCACTCGGTGCTGGT
DmtB1 N159A-FP	CGCAAGGTGCTGCGCGCACACCTGGCCGGGG
DmtB1 N159A-RP	CCCCGGCCAGGTGTGCGCGCAGCACCTTGCG
DmtB1 V205W-FP	TCTACCAGCACCGATGGGAGTTCATCGACCG
DmtB1 V205W-RP	CGGTCGATGAACTCCCATCGGTGCTGGTAGA
DmtB1 V205M-FP	TCTACCAGCACCGAATGGAGTTCATCGACC
DmtB1 V205M-RP	GGTCGATGAACTCCATTCGGTGCTGGTAGA
DmtB1 M65F-FP	GGCGGGTGGACGTCTTTATCCCCGACTCGGC
DmtB1 M65F-RP	GCCGAGTCGGGGATAAAGACGTCCACCCGCC
DmtB1 K155A-FP	ACCGGGTAGTACGCGCAGTGCTGCGCAACCA
DmtB1 K155A-RP	TGGTTGCGCAGCACTGCGCGTACTACCCGGT
DmtB1 F184A-FP	TCGCCGAGACGCCCGCACTGATCAACACGCC
DmtB1 F184A-RP	GGCGTGTTGATCAGTGCGGGCGTCTCGGCGA
DmtB1 K155E-FP	ACCGGGTAGTACGCGAAGTGCTGCGCAACCAC
DmtB1 K155E-RP	GTGGTTGCGCAGCACTTCGCGTACTACCCGGT
DmtB1 K155R-FP	ACCGGGTAGTACGCCGAGTGCTGCGCAACCAC
DmtB1 K155R-RP	GTGGTTGCGCAGCACTCGGCGTACTACCCGGT
DmtB1 K155L-FP	ACCGGGTAGTACGCCTGGTGCTGCGCAACCAC
DmtB1 K155L-RP	GTGGTTGCGCAGCACCAGGCGTACTACCCGGT
DmtB1 H203A-FP	CCGCCATCTACCAGGCACGAGTGGAGTTCAT
DmtB1 H203A-RP	ATGAACTCCACTCGTGCCTGGTAGATGGCGG
DmtB1 M65V-FP	GGCGGGTGGACGTCGTTATCCCCGACTCGGC
DmtB1 M65V-RP	GCCGAGTCGGGGATAACGACGTCCACCCGCC
DmtB1 L185F-FP	CCGAGACGCCCTTCTTTATCAACACGCCCC
DmtB1 L185F-RP	GGGGCGTGTTGATAAAGAAGGGCGTCTCGG
DmtB1 A199S-FP	TGGACAGCTCGACCAGCATCTACCAGCACCG
DmtB1 A199S-RP	CGGTGCTGGTAGATGCTGGTCGAGCTGTCCA
DmtB1 A199N-FP	TGGACAGCTCGACCAATATCTACCAGCACCG
DmtB1 A199N-RP	CGGTGCTGGTAGATATTGGTCGAGCTGTCCA
DmtB1 V152M-FP	AGAGCTACCACCGGATGGTACGCAAGGTGC
DmtB1 V152M-RP	GCACCTTGCGTACCATCCGGTGGTAGCTCT
DmtB2 V74M-FP	CCGGATCGACGTCATGATCCCCGACAGCGCG
DmtB2 V74M-RP	CGCGCTGTCGGGGATCATGACGTCGATCCGG
DmtB2 L194F-FP	GGAGACCCCGCTGTTTATCGGTGCCCCGGAC
DmtB2 L194F-RP	GTCCGGGGCACCGATAAACAGCGGGGTCTCC
DmtB2 I214M-FP	TTTACCACAAGCGCATGGACTTCATCGACG
DmtB2 I214M-RP	CGTCGATGAAGTCCATGCGCTTGTGGTAAA

[a] Underlined letters represent mutated residues. The primer pairs of DmtB1 E206A-FP/RP, DmtB1 E206P-FP/RP, DmtB1 N159A-FP/RP, DmtB1 V205W-FP/RP, DmtB1 V205M-FP/RP, DmtB1 M65F-FP/RP, DmtB1 K155A-FP/RP, DmtB1 F184A-FP/RP, DmtB1 K155E-FP/RP, DmtB1 K155R-FP/RP, DmtB1 K155L-FP/RP, DmtB1 H203A-FP/RP, DmtB1 M65V-FP/RP, DmtB1 L185F-FP/RP, DmtB1 A199S-FP/RP, DmtB1 A199N-FP/RP, DmtB1 V152M-FP/RP were used for constructing mutants of DmtB1 E206A, E206P, N159A, V205W, M65F, K155A, F184A, K155E, K155R, K155L, H203A, M65V, L185F, A199S, A199N and V152M, respectively. The primer pairs of DmtB2 V74M-FP/RP, DmtB2 L194F-FP/RP, and DmtB2 I214M-FP/RP, were used for constructing variants of DmtB2 V74M, L194F, and I214M, respectively.

Supplementary Table 4. The primer pairs used for DmtA1 site-directed mutagenesis[a]

Name	Sequence (5'-3')
DmtA1 Y33F-FP	GCTGGACCATCGCGTTCGTGCTGGCGGTACG
DmtA1 Y33F-RP	CGTACCGCCAGCACGAACGCGATGGTCCAGC
DmtA1 Y217F-FP	TGCTCGACGTCGCGTTCATCGCCGCCCTCTC
DmtA1 Y217F-RP	GAGAGGGCGGCGATGAACGCGACGTCGAGCA
DmtA1 E60Q-FP	GAACATCTGCTGGCAGTTCCAGTTCGTCTTC
DmtA1 E60Q-RP	GAAGACGAACTGGAACTGCCAGCAGATGTTC
DmtA1 D94N-FP	TCTGGCTCATCGTCAACTGCGGCCTGCTGTAC
DmtA1 D94N-RP	GTACAGCAGGCCGCAGTTGACGATGAGCCAGA
DmtA1 D214N-FP	CCTGCGTCCTGCTCAACGTCGCGTACATCGC
DmtA1 D214N-RP	GCGATGTACGCGACGTTGAGCAGGACGCAGG
DmtA1 Y33A-FP	GCTGGACCATCGCGGCCGTGCTGGCGGTACG
DmtA1 Y33A-RP	CGTACCGCCAGCACGGCCGCGATGGTCCAGC
DmtA1 E60A-FP	GAACATCTGCTGGGCCTTCCAGTTCGTCTTC
DmtA1 E60A-RP	GAAGACGAACTGGAAGGCCCAGCAGATGTTC
DmtA1 Y217A-FP	TGCTCGACGTCGCGGCCATCGCCGCCCTCTC
DmtA1 Y217A-RP	GAGAGGGCGGCGATGGCCGCGACGTCGAGCA
DmtA1 D214A-FP	CCTGCGTCCTGCTCGCCGTCGCGTACATCGC
DmtA1 D214A-RP	GCGATGTACGCGACGGCGAGCAGGACGCAGG
DmtA1 W29A-FP	ATGCGGCCTGGGCGCCACCATCGCGTATGTG
DmtA1 W29A-RP	CACATACGCGATGGTGGCGCCCAGGCCGCAT
DmtA1 N56A-FP	TGGCACTCGCCATGGCCATCTGCTGGGAATTC
DmtA1 N56A-RP	GAATTCCCAGCAGATGGCCATGGCGAGTGCCA
DmtA1 W59A-FP	CCATGAACATCTGCGCCGAATTCCAGTTCGTC
DmtA1 W59A-RP	GACGAACTGGAATTCGGCGCAGATGTTCATGG
DmtA1 C58A-FP	TCGCCATGAACATCGCCTGGGAATTCCAGTTC
DmtA1 C58A-RP	GAACTGGAATTCCCAGGCGATGTTCATGGCGA
DmtA1 D94A-FP	TCTGGCTCATCGTCGCCTGCGGCCTGCTGTAC
DmtA1 D94A-RP	GTACAGCAGGCCGCAGGCGACGATGAGCCAGA
DmtA1 E133A-FP	CCTACGCGGGCATCGCCGTGCTCTCCCGGGAA
DmtA1 E133A-RP	TTCCCGGGAGAGCACGGCGATGCCCGCGTAGG

[a] Underlined letters represent mutated residues. The primer pairs of DmtA1 Y33F-FP/RP, DmtA1 Y217F-FP/RP, DmtA1 E60Q-FP/RP, DmtA1 D94N-FP/RP, DmtA1 D214N-FP/RP, DmtA1 Y33A-FP/RP, DmtA1 E60A-FP/RP, DmtA1 Y217A-FP/RP, DmtA1 D214A-FP/RP, DmtA1 W29A-FP/RP, DmtA1 N56A-FP/RP, DmtA1 W59A-FP/RP, DmtA1 C58A-FP/RP, DmtA1 D94A-FP/RP, and DmtA1 E133A-FP/RP were used for constructing variants of DmtA1 Y33F, Y217F, E60Q, D94N, D214N, Y33A, E60A, Y217A, D214A, W29A, N56A, W59A, C58A, D94A, and E133A, respectively.

Supplementary Table 5. The primer pair used for cosmid library screening

cosmid	Primer pairs used for cosmid library screening (5'-3')
pWLI614	CGGTGATCGGCCCGAAAT<br>CGTCAGCGTGCCCACAGAA

Supplementary Table 6. The primer pairs used for PCR-targeted mutagenesis[a]

gene	Primer pairs used for genes inactivation and overexpression (5'-3')
dmtA1	*dmtA1*MF: CGTGCGACCGCACAGCTCCAAAGCTCCAAGGGGGGACATGattccggggatccgtcgacc
	*dmtA1*MR: CGTCACAGCGAAGCGTTCCTTTCGATGTCCCGGATTTCGGGtgtaggctggagctgcttc
	*dmtA1*CF: CGCTGTGCAATCGAAACG
	*dmtA1*CR: ACCGTCGAAGGGTTGGTGT
	*dmtA1*EF: ATGGTAGACGGCATCCGTATG
	*dmtA1*ER: GCTCTAGATCGTCACAGCGAAGCGTTCC
dmtB1	*dmtB1*MF: GGCCCGAAATCCGGGACATCGAAAGGAACGCTTCGCTGTGattccggggatccgtcgacc
	*dmtB1*MR: CGGGTGACGACGAACGCCTGCTGCTCGTTCCGGCTCAGGGGtgtaggctggagctgcttc
	*dmtB1*CF: CGGTGATCGGCCCGAAAT
	*dmtB1*CR: CGTCAGCGTGCCCACAGAA
	*dmtB1*EF: GTGACGAACACCAACCCTTC
	*dmtB1*ER: GCTCTAGACGCCTGCTGCTCGTTCCG
dmtC1	*dmtC1*MF: CCAGGAAATGGACGCGGCCGAGATCCGCGACCCGCTCCTGattccggggatccgtcgacc
	*dmtC1*MR:GTCGAGTCGGCGGTCAGGGCGAAGACGTCGTGGTCGCAGCGtgtaggctggagctgcttc
	*dmtC1*CF: CCAGCACCGAGTGGAGTTCATC
	*dmtC1*CR: TTACGGACTGCGGGTTACGG
	*dmtC1*EF: ATGACCCGCCAGGAAATGG
	*dmtC1*ER: GCTCTAGACGGGTTACGGACTGCGGGTTA

[a] The restriction site of *Xba*I was underlined. The primer pairs of *dmtA1*MF/MR, *dmtB1*MF/MR, and *dmtC1*MF/MR were used for gene *dmtA1, dmtB1,* and *dmtC1* deletion. The primer pairs of *dmtA1*CF/CR, *dmtB1*CF/CR, and *dmtC1*CF/CR were used for PCR confirmation of the mutants. The primer pairs of *dmtA1*EF/ER, *dmtB1*EF/ER, and *dmtC1*EF/ER were used for *dmtA1, dmtB1,* and *dmtC1* genetic complementation and overexpression experiments.

Supplementary Table 7. The ratios of cWP *vs* cWV and cWL *vs* cWV by DmtB1 and its variants in the *in vivo* and the *in vitro* assays

Proteins	*in vivo*		*in vitro*	
	cWP *vs* cWV (**2** *vs* **3**)	cWL *vs* cWV (**4** *vs* **3**)	cWP *vs* cWV (**2** *vs* **3**)	cWL *vs* cWV (**4** *vs* **3**)
DmtB1-WT	1:13.6	1:10	1:518.8	1:33.3
DmtB1-L185F	22.8:1	13:1	1:4.3	1:4.1
DmtB1-V205M	1:16.4	1.1:1	1:88.6	1:5

Supplementary Table 8. LC–MS/MS conditions for the analytes by MRM in positive ionization mode

Compounds	Precursor ion	Product ion	Fragmentor	Collision energy
cWL/cWI	300.18	170.04	100	20
cWL/cWI	300.18	130.11	100	22
cWV	286.18	170.04	100	20
cWV	286.18	130.11	100	22
cWP	284.15	170.04	100	20
cWP	284.15	130.11	100	25

Supplementary Table 9. ^{1}H and ^{13}C NMR data of pre-drimentine compounds (**6-8**, and **13**) in DMSO-d_6 [a]

Position	pre-drimentine C, **6**		pre-drimentine G, **7**		pre-drimentine A, **8**		pre-drimentine J, **13**	
	δ_H (*J* in Hz)	δ_C	δ_H (*J* in Hz)	δ_C	δ_H (*J* in Hz)	δ_C	δ_H (*J* in Hz)	δ_C
1		166.1		165.2		166.5		165.2
2								
3	5.16 (s)	78.4	5.25 (s)	78.5	5.19 (s)	78.6	5.25 (s)	78.5
4	6.45 (s)		6.47 (s)		6.45 (s)		6.45 (s)	
5		149.8		150.1		149.9		150.1
6	6.54 (d, 7.5)	108.7	6.52 (d, 7.2)	108.4	6.53 (d, 7.2)	108.6	6.51 (d, 7.2)	108.4
7	6.96 (t, 7.5)	128.1	6.96 (td, 7.2, 0.6)	128.1	6.96 (td, 7.2, 0.6)	128.0	6.95 (td, 7.2, 0.6)	128.1
8	6.60 (t, 7.5)	117.6	6.59 (td, 7.2, 0.6)	117.5	6.60 (td, 7.2, 0.6)	117.5	6.61 (td, 7.2, 0.6)	117.6
9	7.12 (d, 7.5)	123.1	7.11 (d, 7.2)	123.1	7.12 (d, 7.2)	123.1	7.11 (d, 7.2)	123.2
10		131.3		131.3		131.4		131.4
11		55.2		54.7		54.7		54.8
12	2.13 (dd, 13.0, 11.5)<br>2.46 (dd, 13.0, 6.5)	39.8	2.07 (t, 12.0)<br>2.42 (dd, 12.0, 6.0)	39.7	2.12 (t, 12.0)<br>2.44 (dd, 12.0, 6.0)	38.5	2.07 (t, 12.0)<br>2.42 (dd, 12.0, 6.0)	39.3
13	4.02 (m)	59.7	3.86 (ddd, 11.4, 6.0, 1.2)	57.8	3.93 (ddd, 11.4, 6.6, 1.2)	58.2	3.85 (ddd, 10.8, 6.0, 1.2)	57.7
14		165.4		169.3		169.5		169.1
15			7.92 (s)		7.98 (s)		7.91 (s)	
16	4.24 (m)	59.8	3.92 (bt, 1.8)	59.5	4.02 (t, 6.0)	52.6	3.96 (bt, 1.8)	59.2
17	2.36 (m)	35.6	2.35 (d, 7.2)	35.7	2.34 (d, 7.8)	35.5	2.35 (m)	35.7
18	5.11 (t, 7.5)	119.2	5.11 (t, 7.2)	119.1	5.12 (t, 7.2)	119.2	5.11 (t, 7.2)	119.1
19		137.4		137.7		137.6		137.8
20	1.94 (m)	39.4	1.93 (m)	39.4	1.94 (m)	39.4	1.93 (m)	39.4
21	2.00 (m)	26.2	1.99 (m)	26.2	2.00 (m)	26.2	1.99 (m)	26.3
22	5.05 (m)	123.5	5.05 (m)	123.9	5.05 (m)	123.9	5.05 (m)	123.9
23		134.4		134.5		134.4		134.5
24	1.91 (m)	39.3	1.92 (m)	39.2	1.93 (m)	39.2	1.92 (m)	39.2
25	2.00 (m)	26.0	2.01 (m)	26.1	2.01 (m)	26.0	2.01 (m)	26.2
26	5.05 (m)	123.9	5.05 (m)	123.9	5.05 (m)	123.9	5.05 (m)	124.0
27		130.6		130.6		130.6		130.7
28	1.62 (s)	25.5	1.62 (s)	25.5	1.63 (s)	25.5	1.62 (s)	25.5
29	1.54 (s)	17.6	1.54 (s)	17.6	1.55 (s)	17.6	1.54 (s)	17.6
30	1.54 (s)	15.8	1.54 (s)	15.8	1.55(s)	15.8	1.55 (s)	15.8
31	1.47 (s)	16.1	1.49 (s)	16.2	1.49 (s)	16.1	1.50 (s)	16.2
32	1.89 (m), 2.12 (m)	27.3	2.32 (m)	28.4	1.38 (m)1.76 (m)	38.1	2.02 (m)	35.6
33	1.89 (m), 1.78 (m)	22.6	0.86 (d, 7.2)	16.5	1.88 (m)	24.0	1.37 (m), 1.28 (m)	24.1
34	3.34 (m)	44.6	0.99 (d, 7.2)	18.1	0.84 (t, 7.2)	22.0	0.83 (t, 7.2)	12.3
35					0.84 (t, 7.2)	22.8	0.96 (d, 7.2)	14.9

[a] ^{1}H NMR data was collected at 500 MHz for compound **6**, and at 600 MHz for compounds **7**-**8** and **13**. ^{13}C NMR data was collected at 125 MHz for compound **6**, and at 150 MHz for compounds **7**-**8** and **13**.

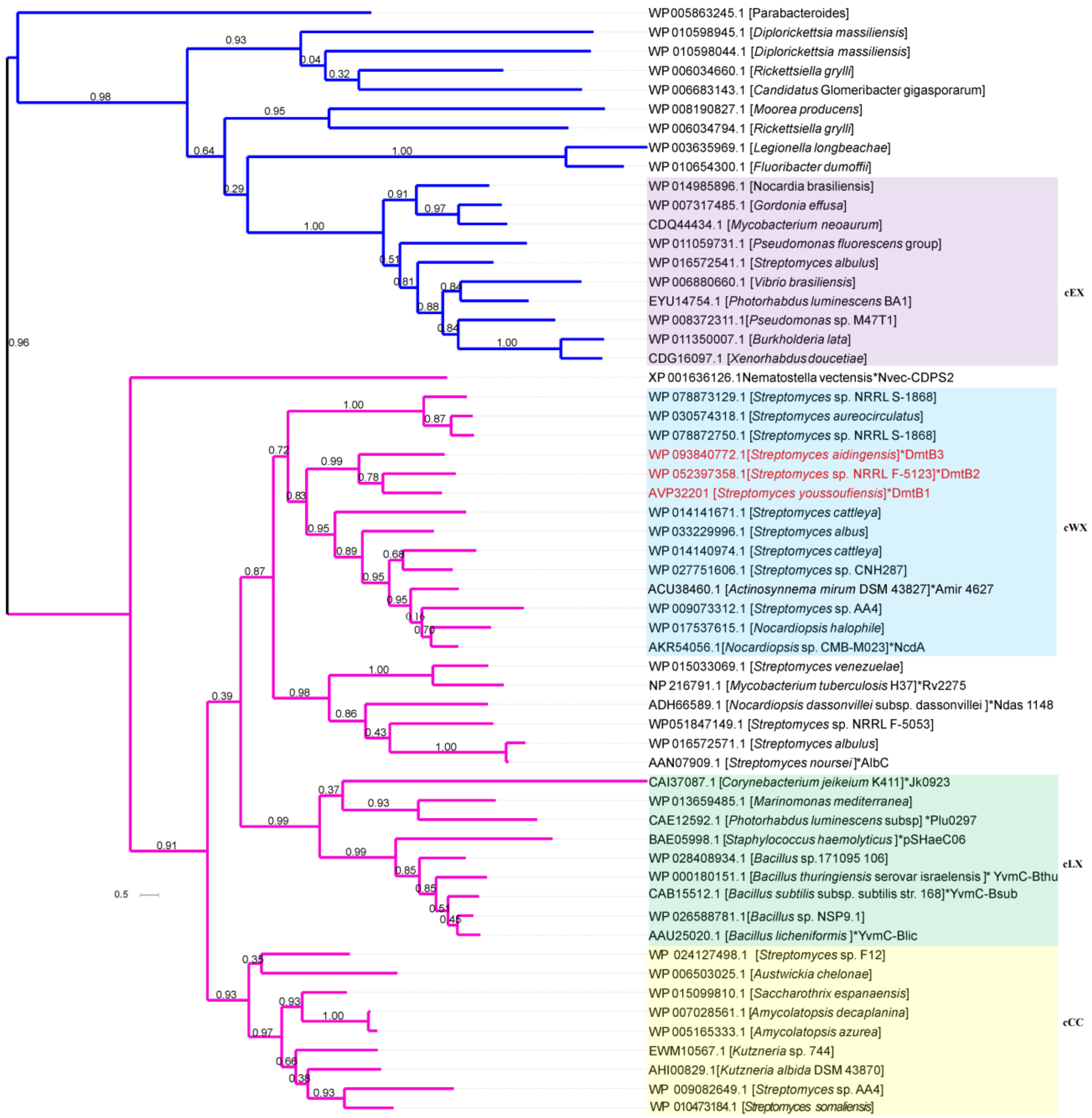

Supplementary Figure 1. Phylogenetic analysis of DmtB1-3 with other selected characterized CDPSs. A phylogenetic tree was generated using PhyML (v3.0)[1] program integrated into Seaview[2] based on the maximum-likelihood method with LG substitution model and NNI tree searching operation. Numbers at each branch point was assessed using the aLRT test (SH-Like).The iTOL software[3] was used for graphical representation and edition of the phylogenetic tree. CDPSs are named by their protein accession numbers in GenBank and their origins are shown in bracket. Two main branches corresponding to the NYH and XYP subfamilies are in fuchsia and blue, respectively.

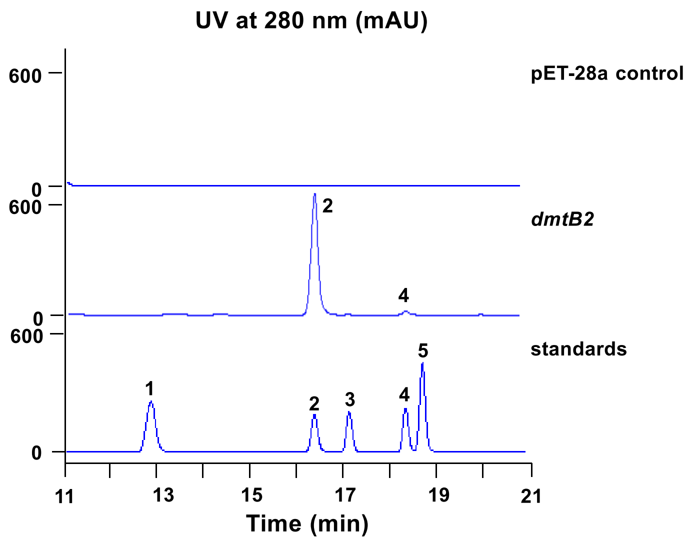

Supplementary Figure 2. HPLC traces of culture supernatants of *E. coli* cells expressing DmtB2.

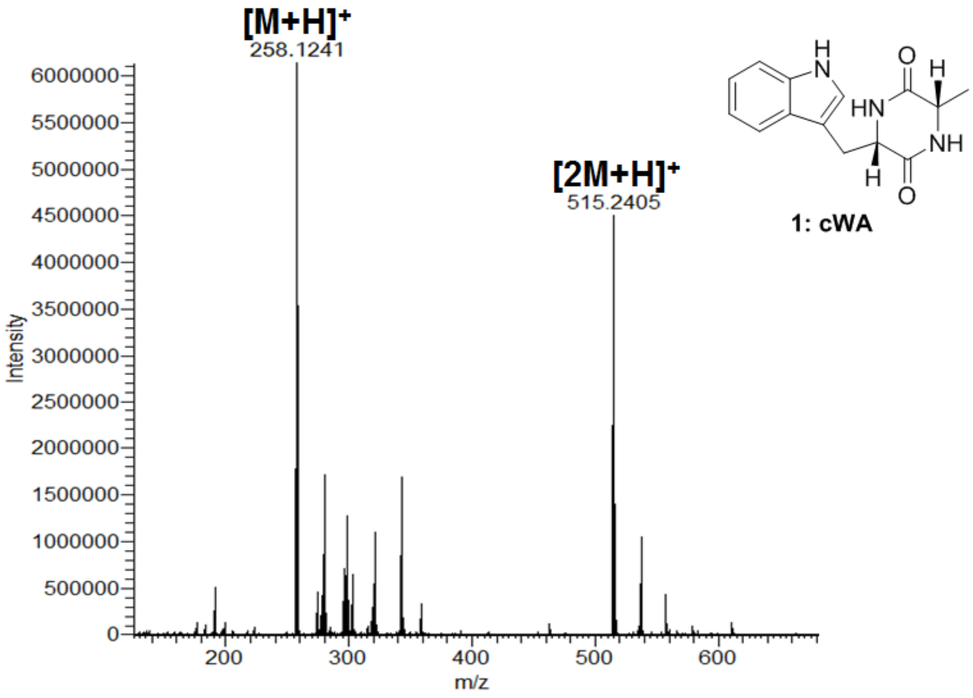

Supplementary Figure 3. HR-ESIMS spectrum of compound **1**.

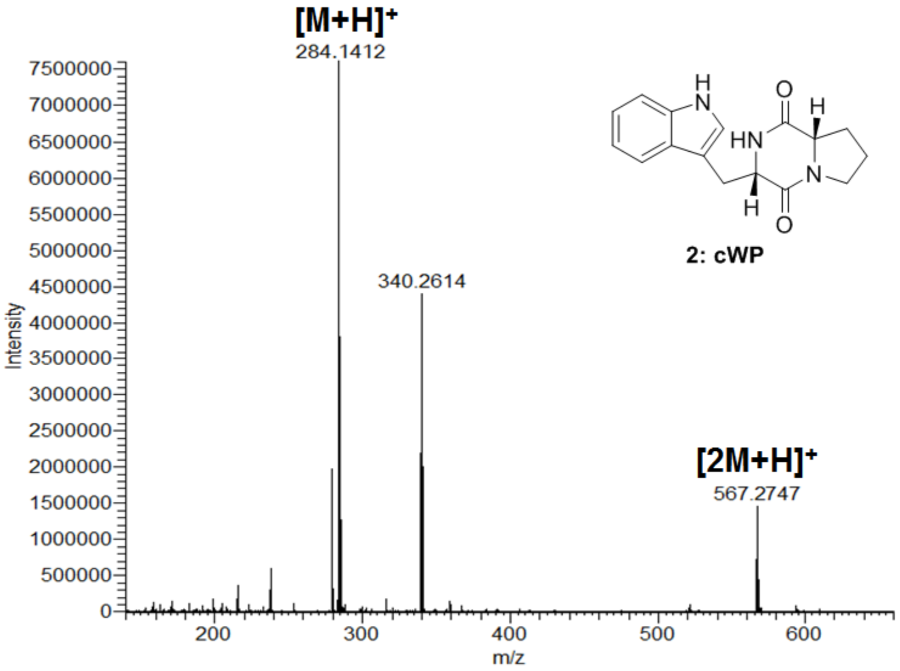

Supplementary Figure 4. HR-ESIMS spectrum of compound **2**.

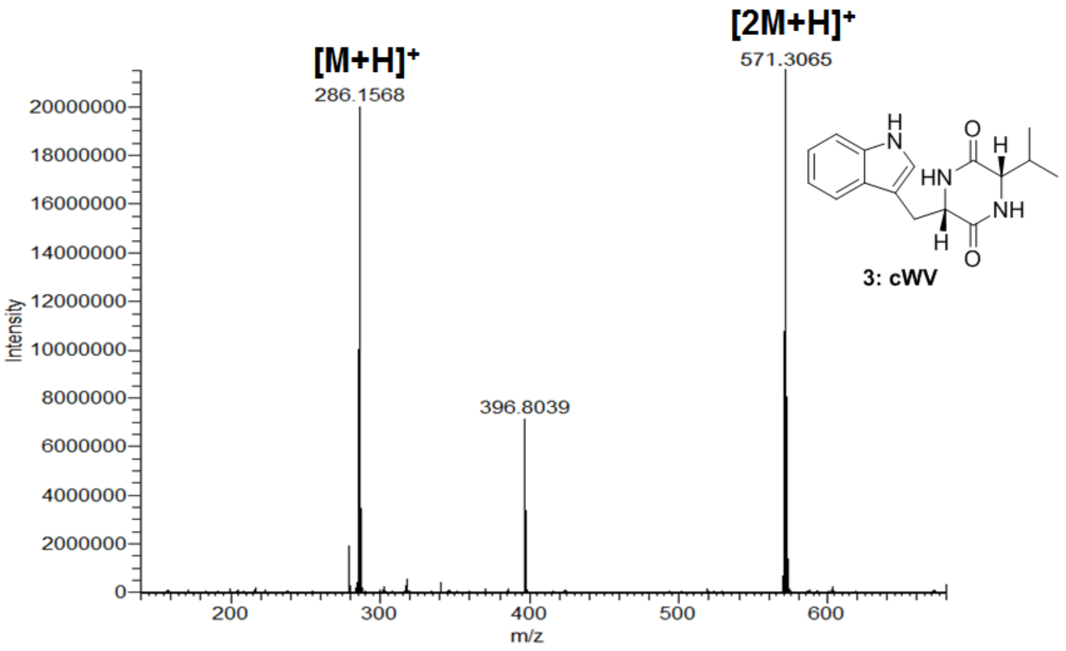

Supplementary Figure 5. HR-ESIMS spectrum of compound **3**.

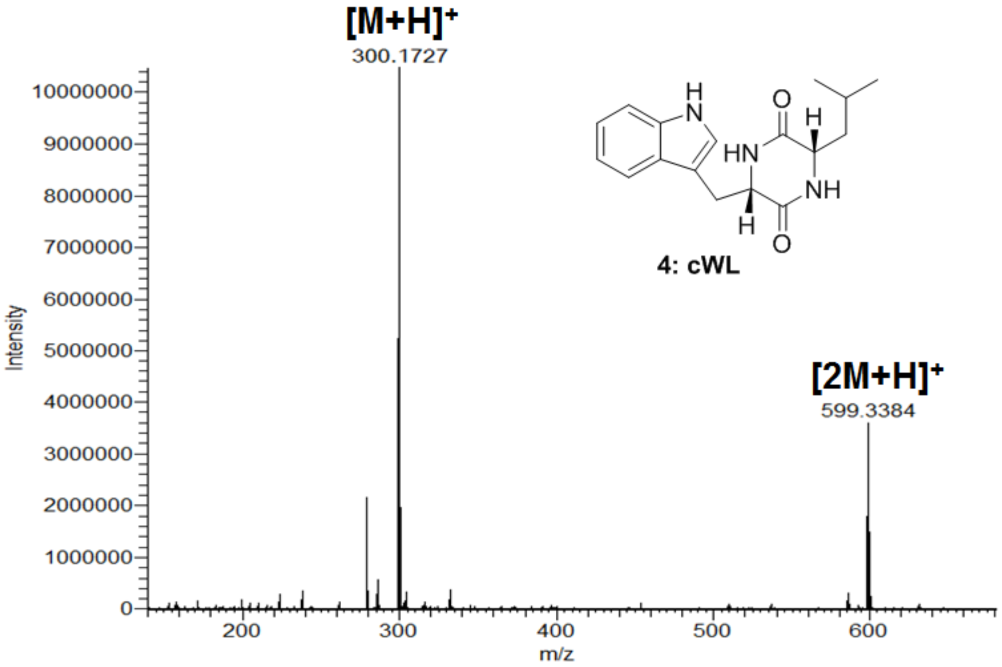

Supplementary Figure 6. HR-ESIMS spectrum of compound **4**.

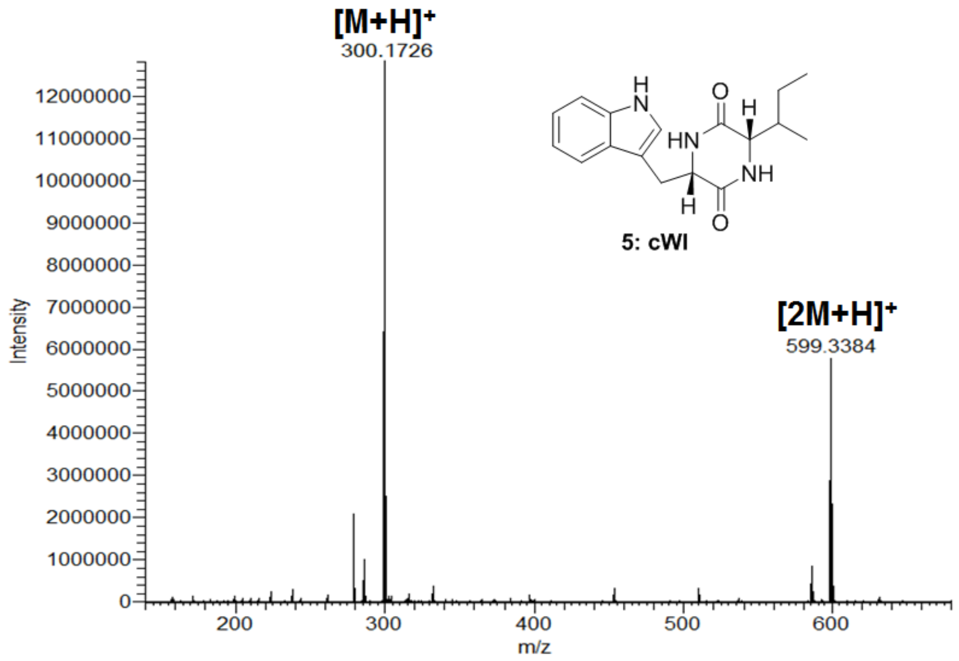

Supplementary Figure 7. HR-ESIMS spectrum of compound **5**.

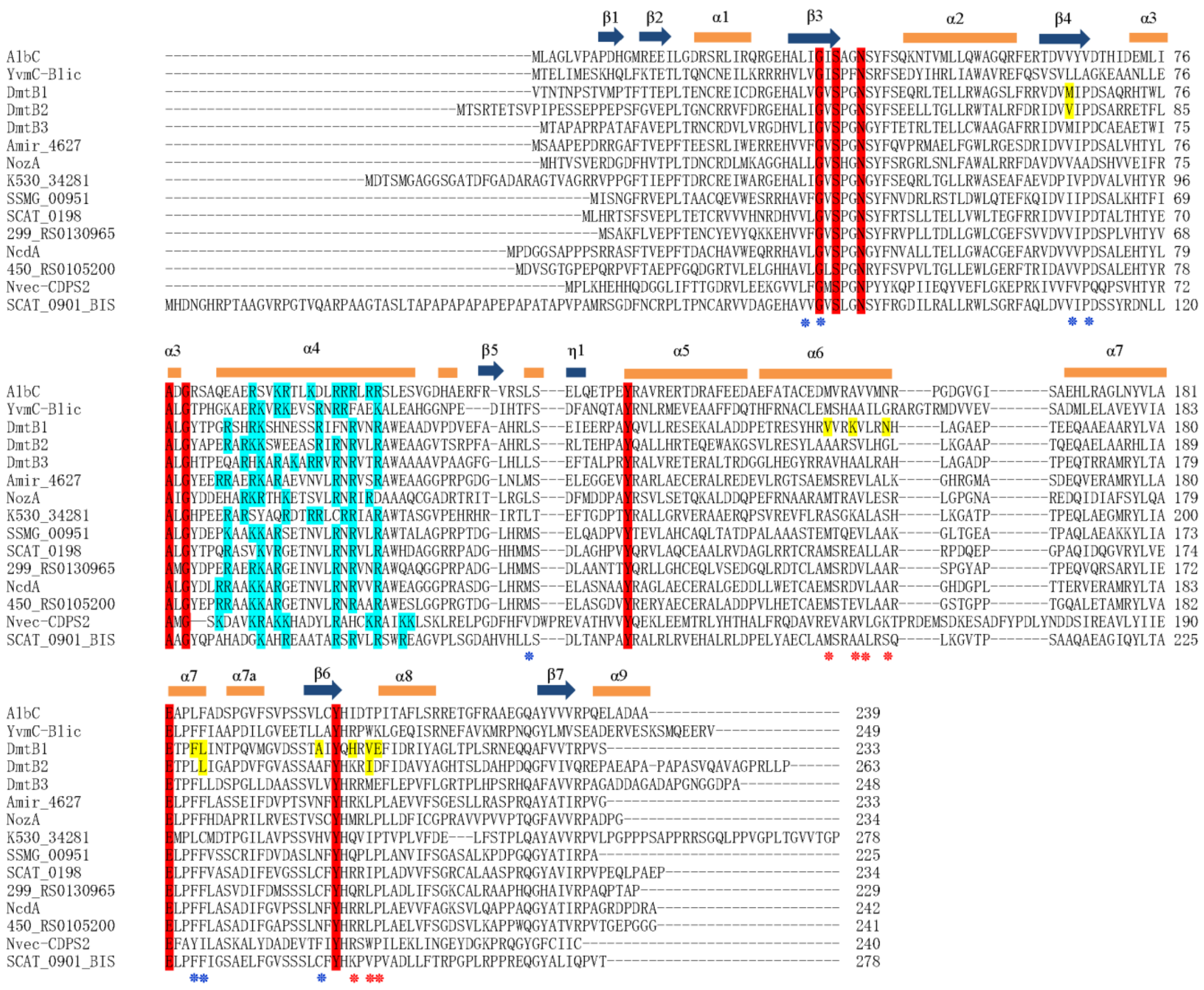

Supplementary Figure 8. Multiple-sequence alignments of DmtB1-3 with selected NYH subfamily CDPSs. The secondary structural elements of AlbC are indicated above the alignment. Residues conserved among all aligned sequences are highlighted in a red background; residues subjected to mutagenesis are shown in a yellow background; positively charged residues in helix α4 are highlighted in a cyan background. Amino acids constituting the two binding pockets P1 and P2 are indicated with blue and red stars, respectively, under the alignment. The origins of the CDPSs are as follows: AlbC (AAN07909.1) from *Streptomyces noursei*, YvmC-Blic (AAU25020.1) from *Bacillus licheniformis*, DmtB1 (AVP32201) from *Streptomyces youssoufiensis* OUC6819, DmtB2 (WP_052397358.1) from *Streptomyces* sp. NRRL F-5123, DmtB3 (WP_093840772.1) from *Streptomyces aidingensis* CGMCC 4.5739, Amir_4627 (ACU38460.1) from *Actinosynnema mirum*, NozA (AKR54045.1) and NcdA (AKR54056.1) from *Nocardiopsis* sp. CMB-M0232, K530_34281 (WP_016576960) from *Streptomyces albulus*, SSMG_00951 (WP_009073312) from *Streptomyces* sp. AA4, SCAT_0198 (WP_014140974) from *Streptomyces cattleya*, 299_RS0130965 (WP_027751606.1) from *Streptomyces* sp. CNH287, 450_RS0105200 (WP_017537615.1) from *Nocardiopsis halophila*, Nvec-CDPS2 (XP_001636126.1) from *Nematostella vectensis* and SCAT_0901_BIS (WP_014141671) from *Streptomyces cattleya*.

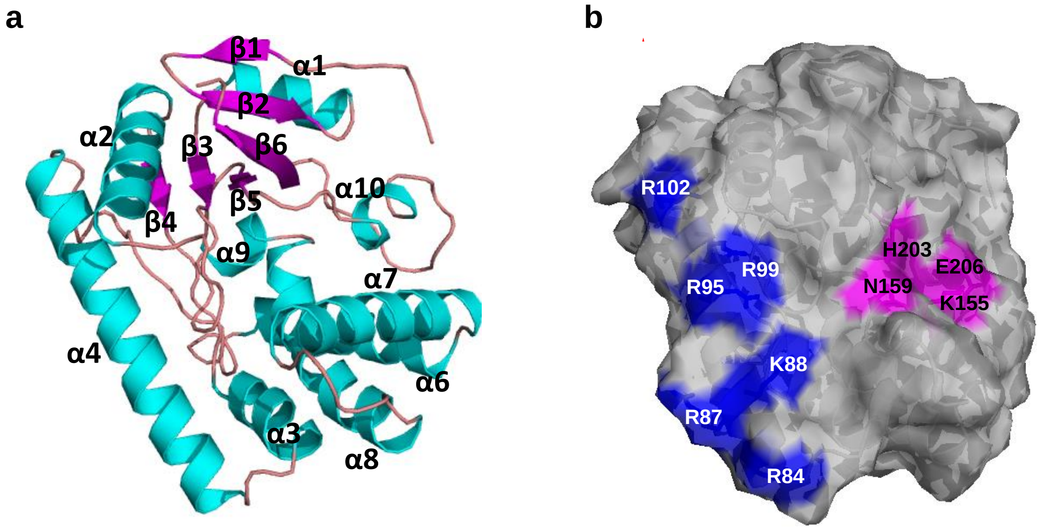

Supplementary Figure 9. Structural model of DmtB1. (**a**) Overall structure of DmtB1 in cartoon mode, with α helices, β-strands, and loops colored in cyan, magenta and wheat, respectively. The secondary-structural elements are numbered according to YvmC[4] numbering. The model was generated by using I-TASSER On-line Server[5,6]. The TM score for this model is 0.94 ± 0.06, indicating a correctly predicted overall topology and a high degree of structural homology to the template, which is additionally confirmed by the low rmsd of 2.6 ± 1.9 Å. (**b**) The overall structure of DmtB1 in surface mode. The putative basic patch of DmtB1 is colored in blue; the charged residues located in pocket 2 are indicated in magenta.

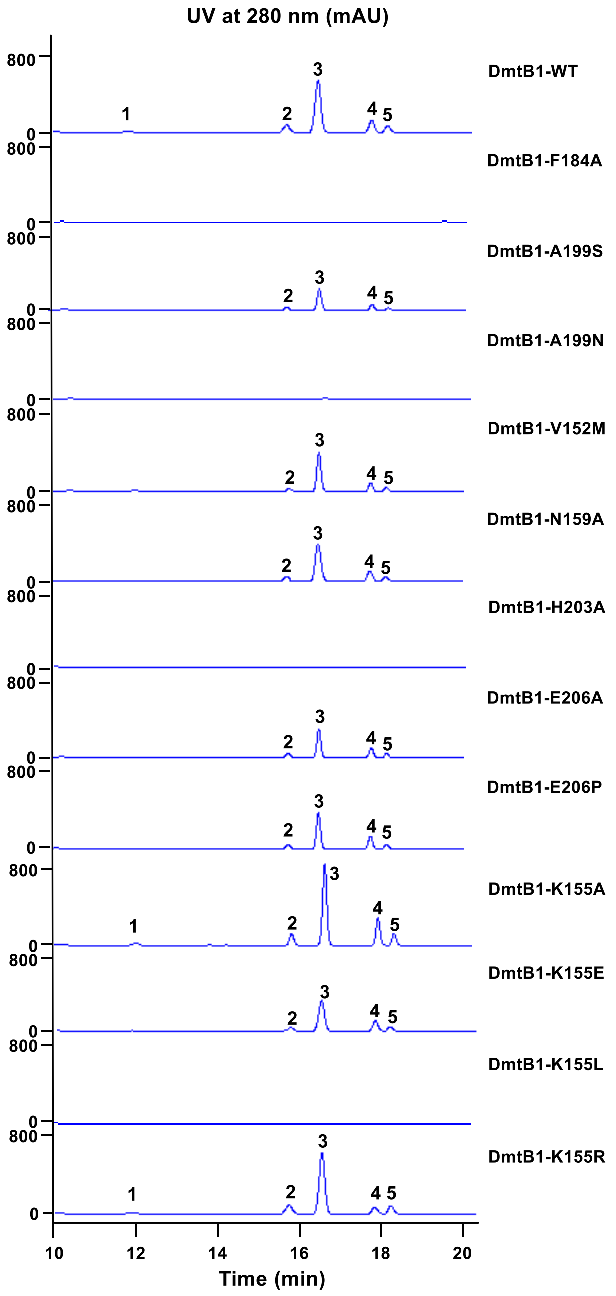

Supplementary Figure 10. HPLC traces of the products formation by DmtB1and its variants. For the residues in the pocket P1, the variant F184A was completely inactive (Supplementary Fig. 10, Fig. 4c); when A199 was substituted with Ser or Asn, both variants displayed defects to synthesize any of the five DKPs by moderate (A199S) and significant (A199N) degrees. For the residues in the pocket P2, the variants of V152M, N159A, E206A and E206P showed moderate defects on the formation of all the five DKPs (Supplementary Fig. 10, Fig. 4c); no expression of the variant H203A was detected indicating this substitution might cause a structural destabilization. Substitution of K155 with alanine increased production by about 1.5-fold for the formation of all the five DKPs, indicating the long side chain of Lys probably has negative impact on the entrance of the second substrate (Fig. 4a). The variant K155L had no detectable activity due to its insoluble expression in *E. coli*. No obvious impact was observed for the variant K155R, conversely, activities of the variant K155E decreased by 2-fold, suggesting the negative charge of Glu might impact the binding of the negatively-charged tRNA moiety while the positive charges of Lys and Arg might promote binding.

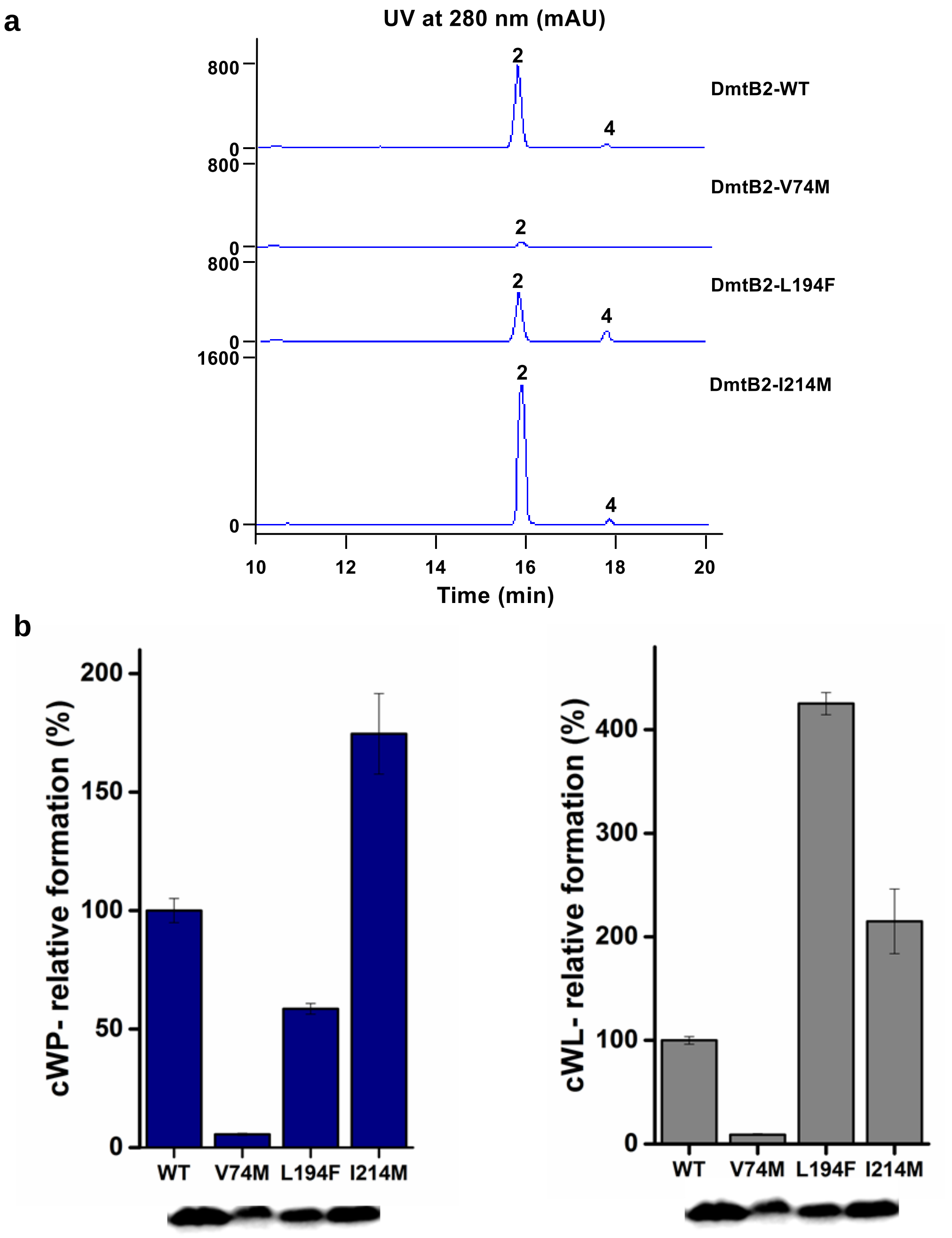

Supplementary Figure 11. Mutagenesis of the residues located in the binding pockets of DmtB2. (**a**) HPLC traces of the products formation by DmtB2 and its variants. (**b**) cWP- (in navy) and cWL-synthesizing activities (in grey) of the wild-type DmtB2 and the variants. The corresponding western blots indicating amounts of the proteins are also shown. Error bars represent ± s.d. of three independent experiments. Substitution of V74 with Met had significant impact on the activity of DmtB2 but the yield of **4** significantly increased by about 4-fold while that of **2** decreased by nearly a half in the variant of L194F (Supplementary Fig. 11a, b). These results further support the observations that substitutions of residues located in the first substrate binding pocket (P1) might affect the incorporation of the second amino acid into DKP. Interestingly, when we substituted I214, corresponding to V205 in DmtB1, with Met, the yields of **2** and **4** were improved by about 2-fold, respectively.

a

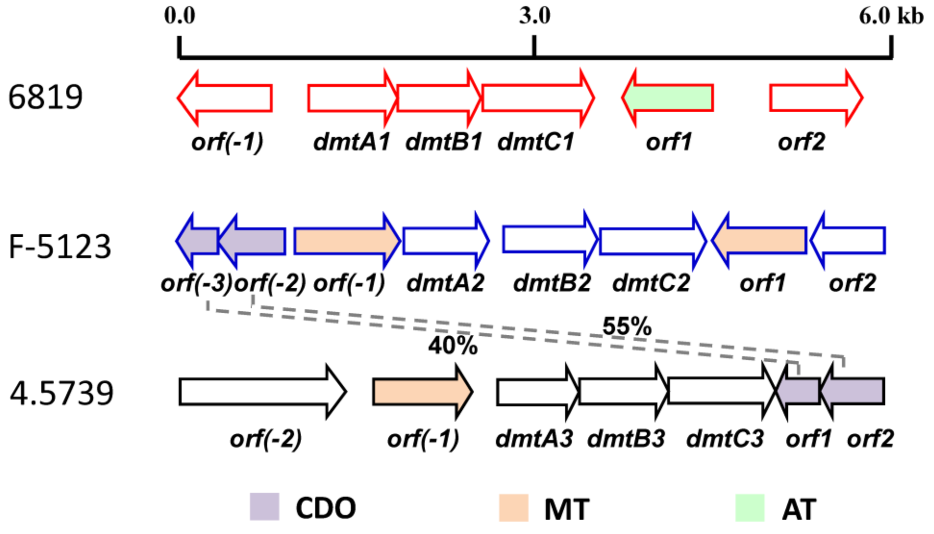

b

Strain	Protein	Size (aa)	Proposed function	Homologs	
				Protein/Organism	Accession no. (Identity/Similarity %)
6819	Orf(-1)	262	pentapeptide repeat-containing protein	ADK70_RS13895 / *Streptomyces rimosus*	WP_053801506.1 (76/83)
	Orf1	253	GCN5 family acetyltransferase	OO69_RS13025 / *Streptomyces* sp. NRRL F-525	WP_033280708.1 (66/76)
	Orf2*	257	threonyl-tRNA ligase	SSTG_04149 / *Streptomyces* sp. e14	EFF93830.1 (67/74, the 1st-70th aa)
F-5123	Orf(-3)	111	CDO subunit	AlbB / *Streptomyces noursei* ATCC 11455	AAN07908.1 (38/55)
	Orf(-2)	189	CDO subunit	AlbA / *Streptomyces noursei* ATCC 11455	AAN07907.1 (42/57)
	Orf(-1)	262	methyltransferase	MitM / *Streptomyces lavendulae*	AAD28459.1 (40/53)
	Orf1	275	methyltransferase	BIV57_15945 / *Streptomyces gilvigriseus*	WP_071657544.1 (81/87)
	Orf2	205	hypothetical protein	BLW85_RS29400 / *Streptomyces misionensis* DSM 40306	WP_074993849.1 (85/91)
4.5739	Orf(-2)	464	cellulose 1,4-beta-cellobiosidase	BMY40_RS00650 / *Streptomyces radiopugnans* CGMCC 4.3519	WP_093654235.1 (55/69)
	Orf(-1)	275	N(5)-glutamine methyltransferase	H181DRAFT_00112 / *Streptomyces* sp. WMMB 714	SCK05782.1 (64/72)
	Orf1	123	CDO subunit	AlbB / *Streptomyces noursei ATCC 11455*	AAN07908.1 (38/46)
	Orf2	177	CDO subunit	AlbA / *Streptomyces noursei* ATCC 11455	AAN07907.1 (42/52)

Supplementary Figure 12. Genetic contexts of *dmt1-3.* (**a**) Comparison of the surrounding genes of *dmt1-3*; **(b)** predicted functions of these surrounding genes. CDO, cyclic dipeptide oxidase AlbA/AlbB homologs; MT, methyltransferase; AT, acetyltransferase; *, Orf2* is a truncated threonyl-tRNA ligase and probably non-functional.

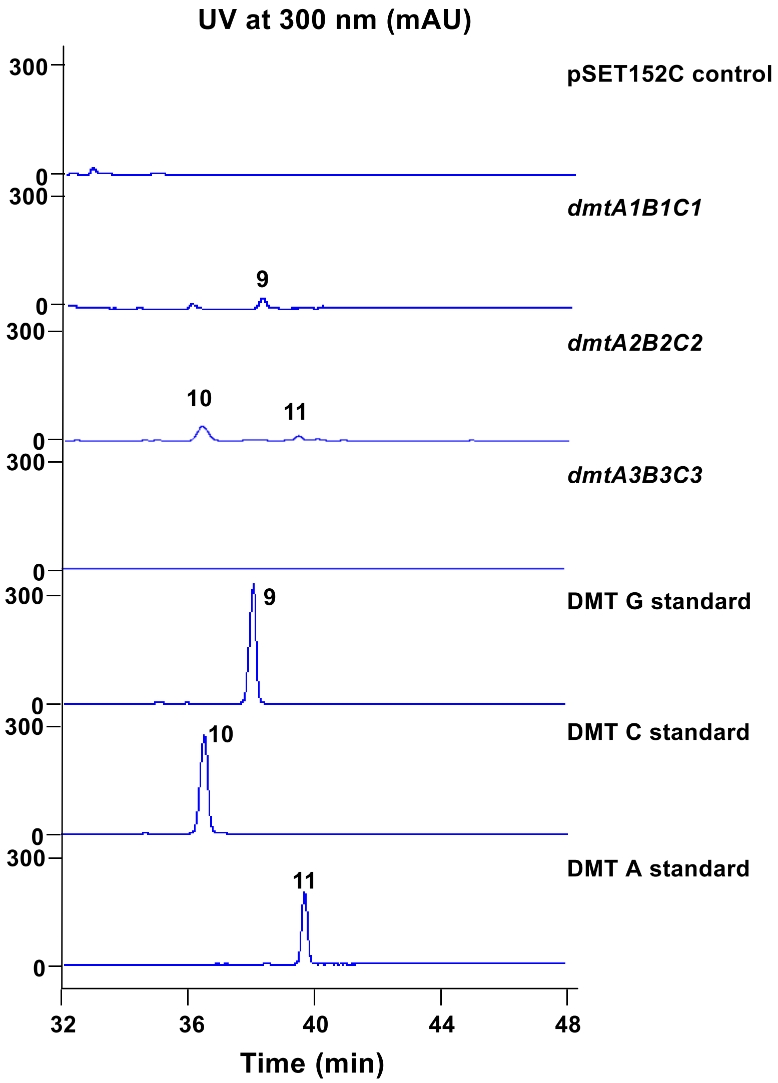

Supplementary Figure 13. Comparison of the *dmt1-3* encoding products in *S. coelicolor* M1146 with DMT standards.

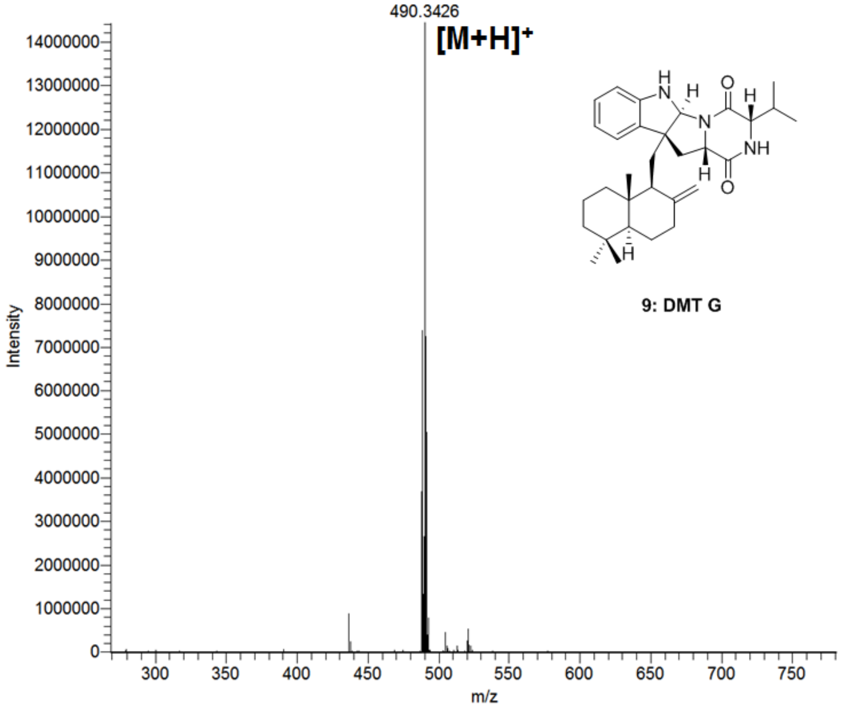

Supplementary Figure 14. HR-ESIMS spectrum of compound **9** (DMT G).

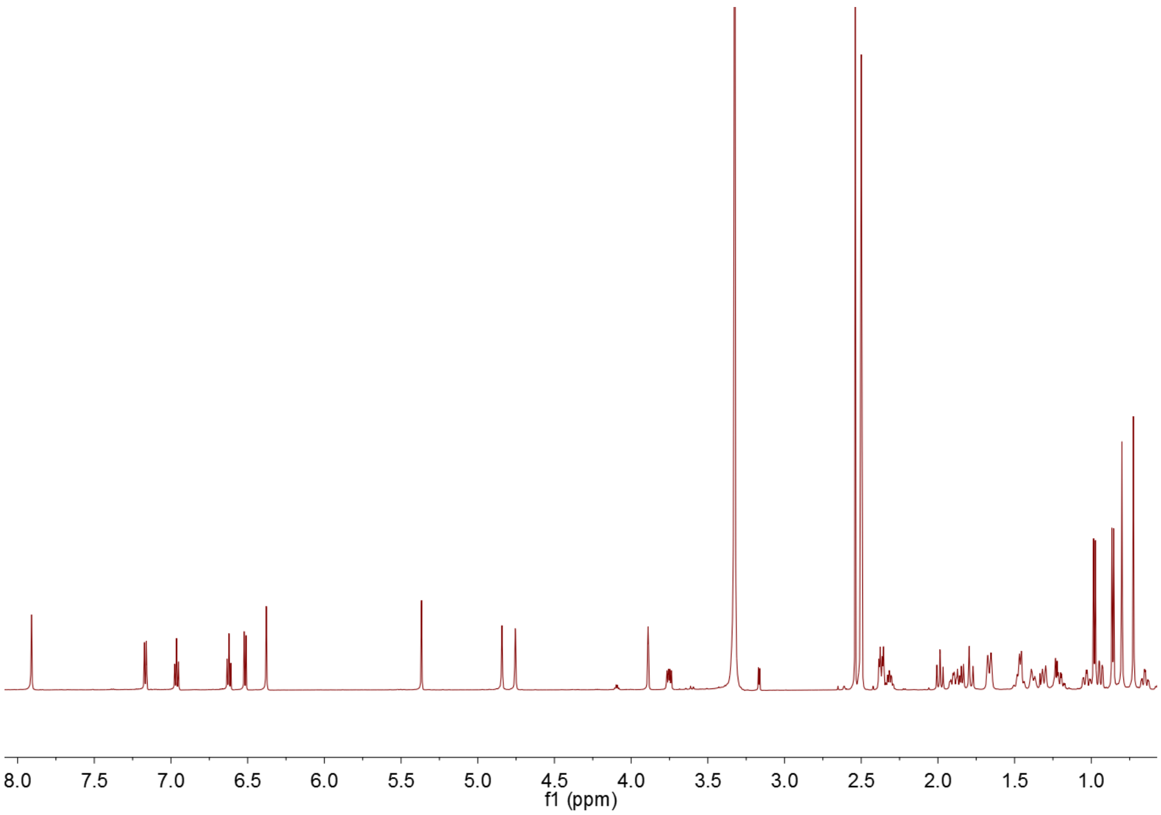

Supplementary Figure 15. ^{1}H NMR spectrum of compound **9** in DMSO-d_6.

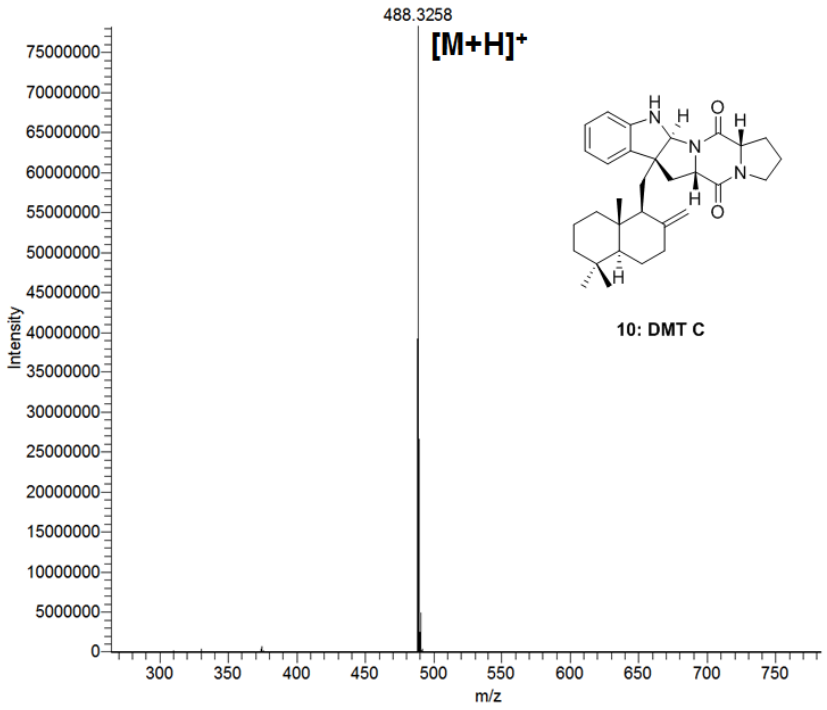

Supplementary Figure 16. HR-ESIMS spectrum of compound **10** (DMT C).

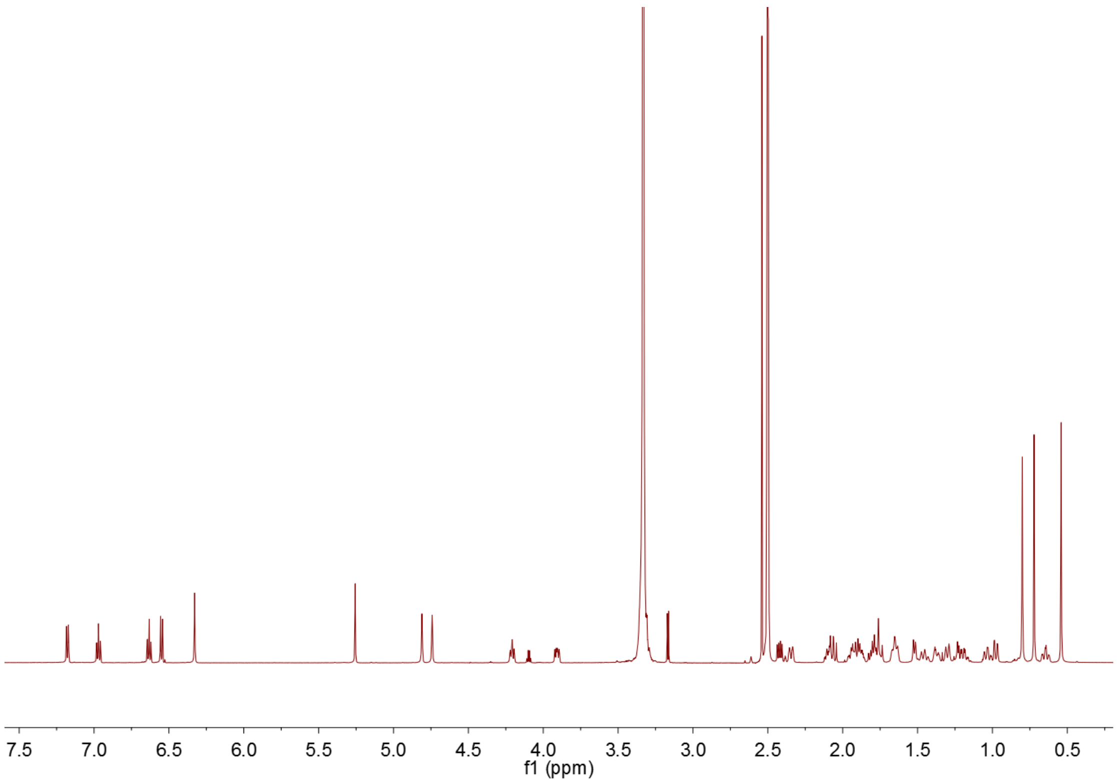

Supplementary Figure 17. ^{1}H NMR spectrum of compound **10** in DMSO-d_6.

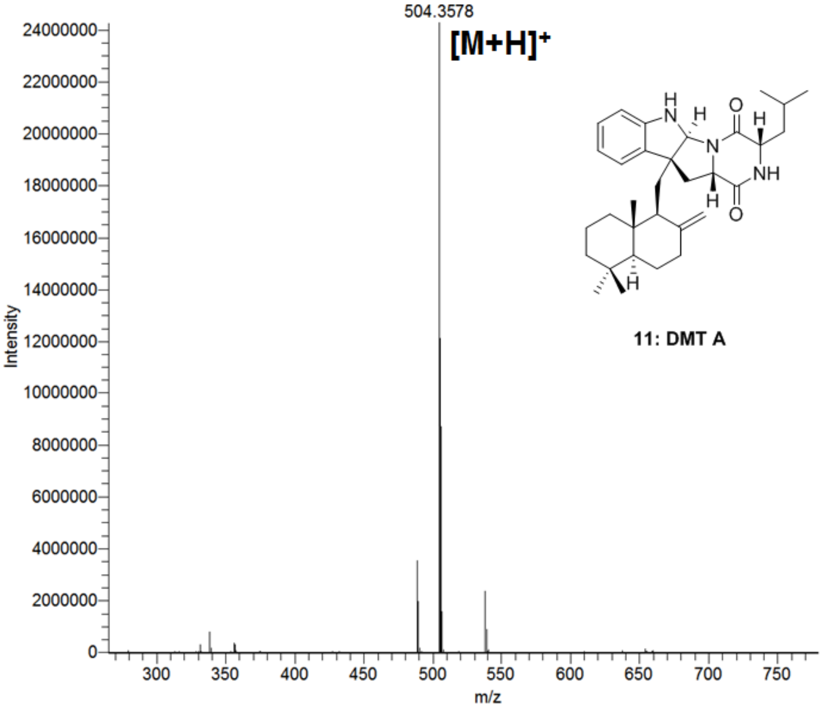

Supplementary Figure 18. HR-ESIMS spectrum of compound **11** (DMT A).

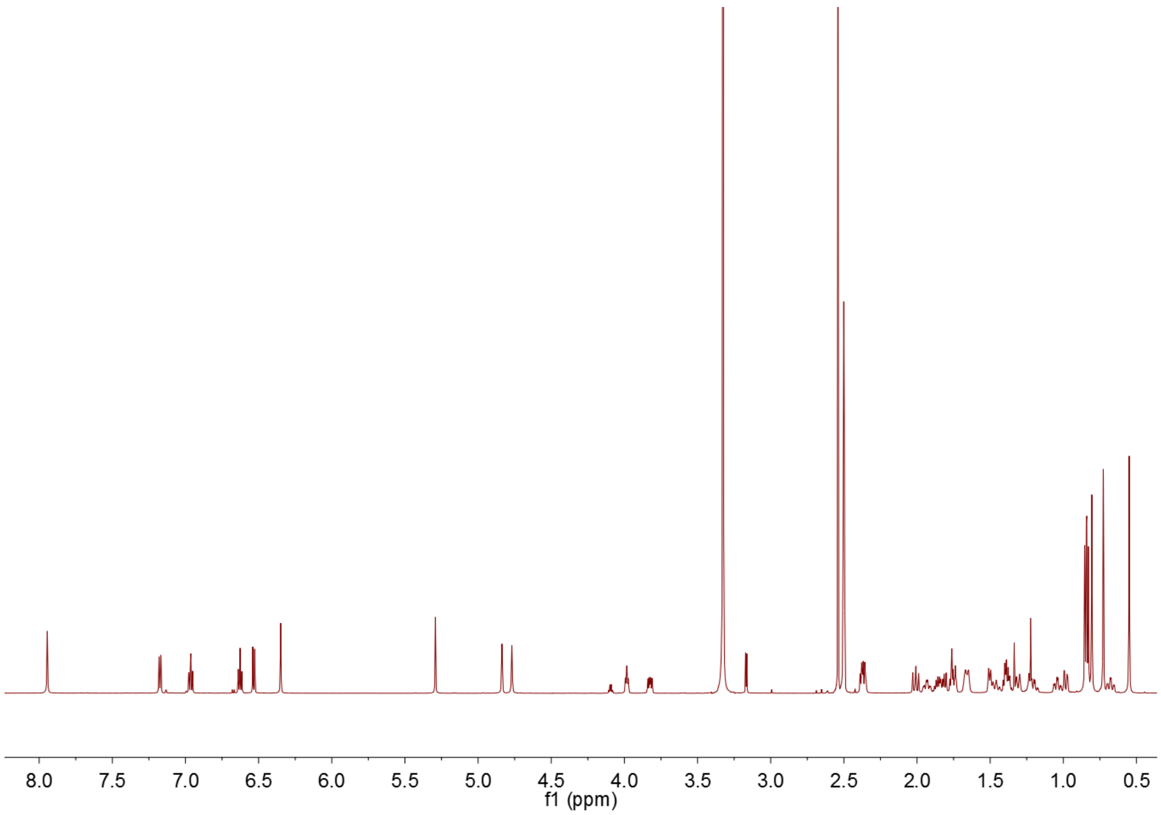

Supplementary Figure 19. ^{1}H NMR spectrum of compound **11** in DMSO-d_6.

a

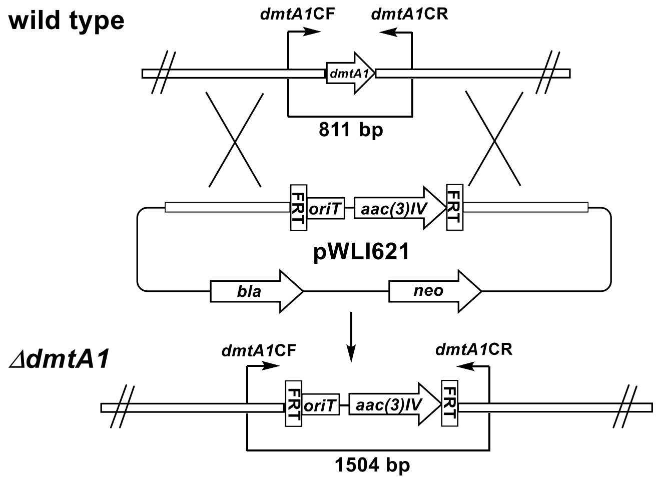

b

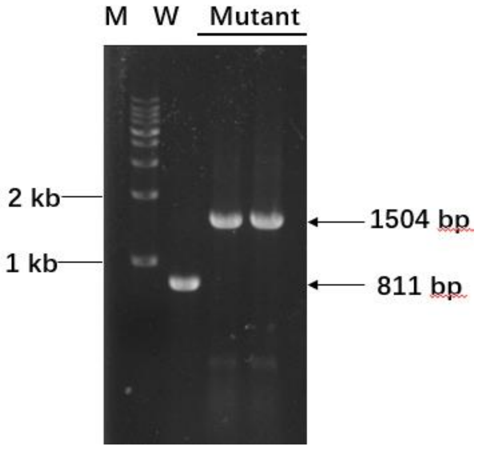

Supplementary Figure 20. Inactivation of *dmtA1*. (**a**) Construction of *dmtA1* gene inactivation mutant. (**b**) PCR confirmation of the double-crossover mutant. M: DNA marker; W: *S. youssoufiensis* OUC6819 wild-type strain; Mutant: *dmtA1* gene inactivation mutant.

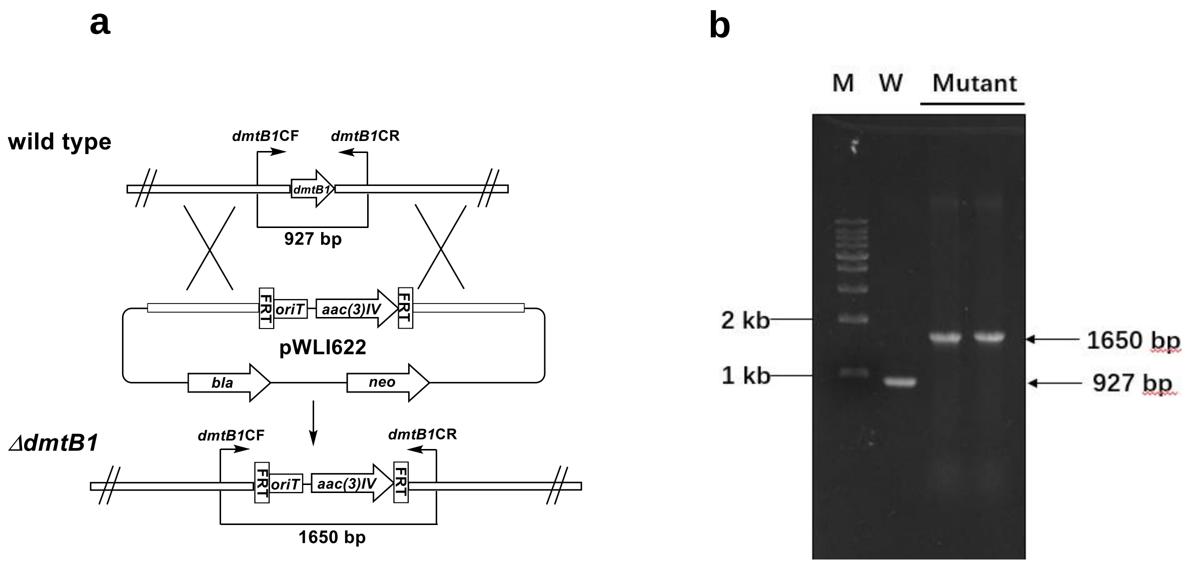

Supplementary Figure 21. Inactivation of *dmtB1*. (**a**) Construction of *dmtB1* gene inactivation mutant. (**b**) PCR confirmation of the double-crossover mutant. M: DNA marker; W: *S. youssoufiensis* OUC6819 wild-type strain; Mutant: *dmtB1* gene inactivation mutant.

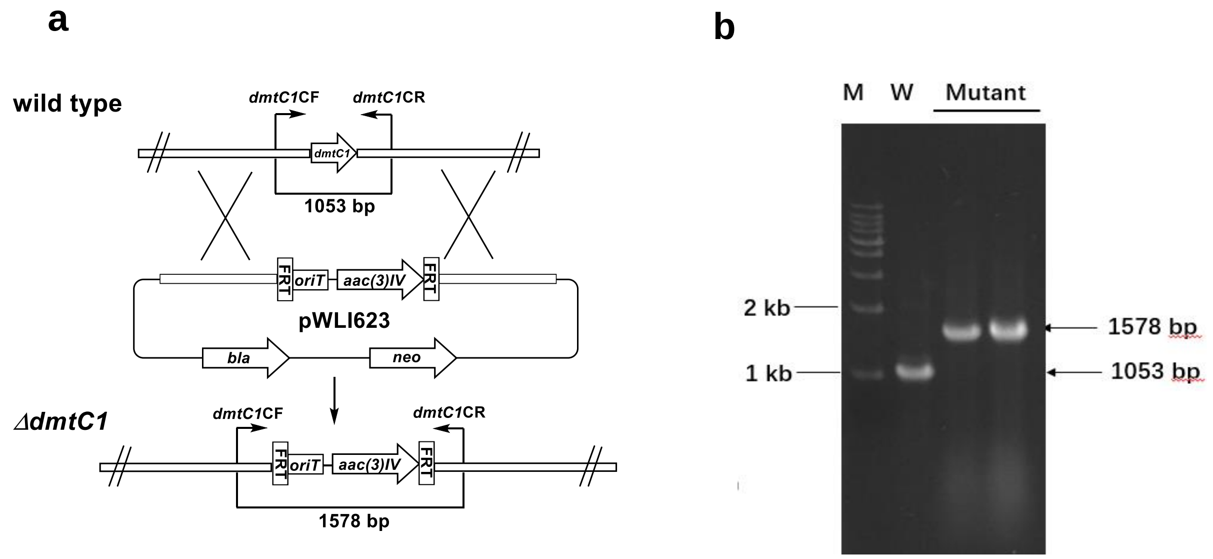

Supplementary Figure 22. Inactivation of *dmtC1*. (**a**) Construction of *dmtC1* gene inactivation mutant. (**b**) PCR confirmation of the double-crossover mutant. M: DNA marker; W: *S. youssoufiensis* OUC6819 wild-type strain; Mutant: *dmtC1* gene inactivation mutant.

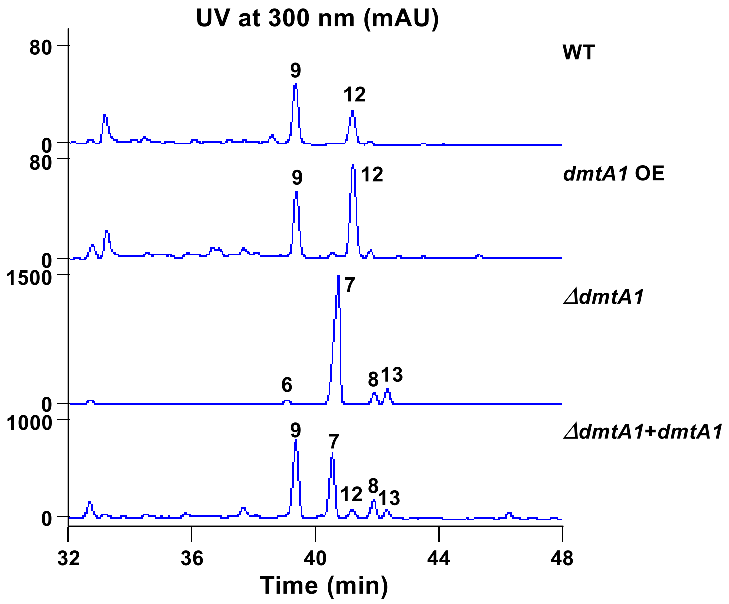

Supplementary Figure 23. *dmtA1* overexpression and genetic complementation of *ΔdmtA1*.

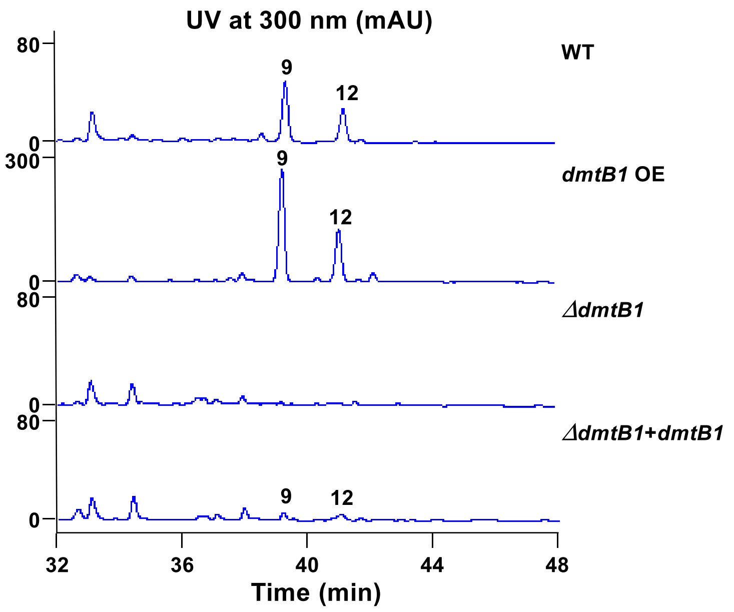

Supplementary Figure 24. *dmtB1* overexpression and genetic complementation of *ΔdmtB1*.

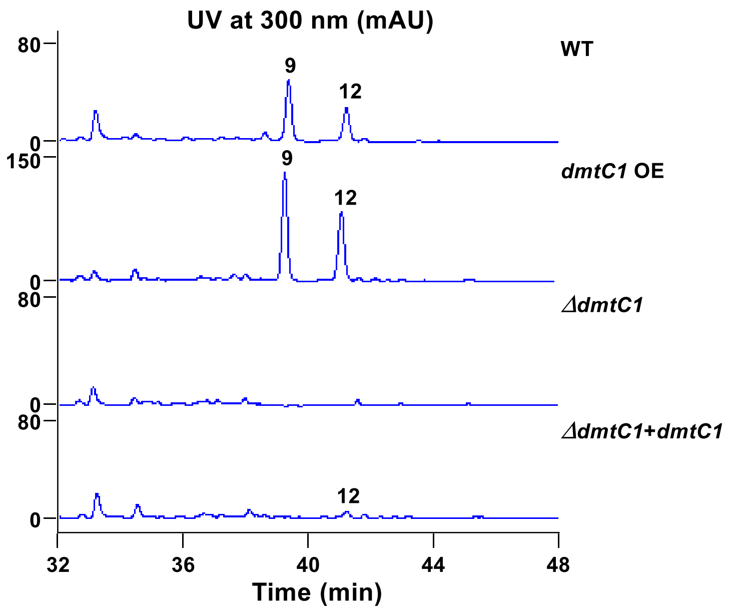

Supplementary Figure 25. *dmtC1* overexpression and genetic complementation of *ΔdmtC1*.

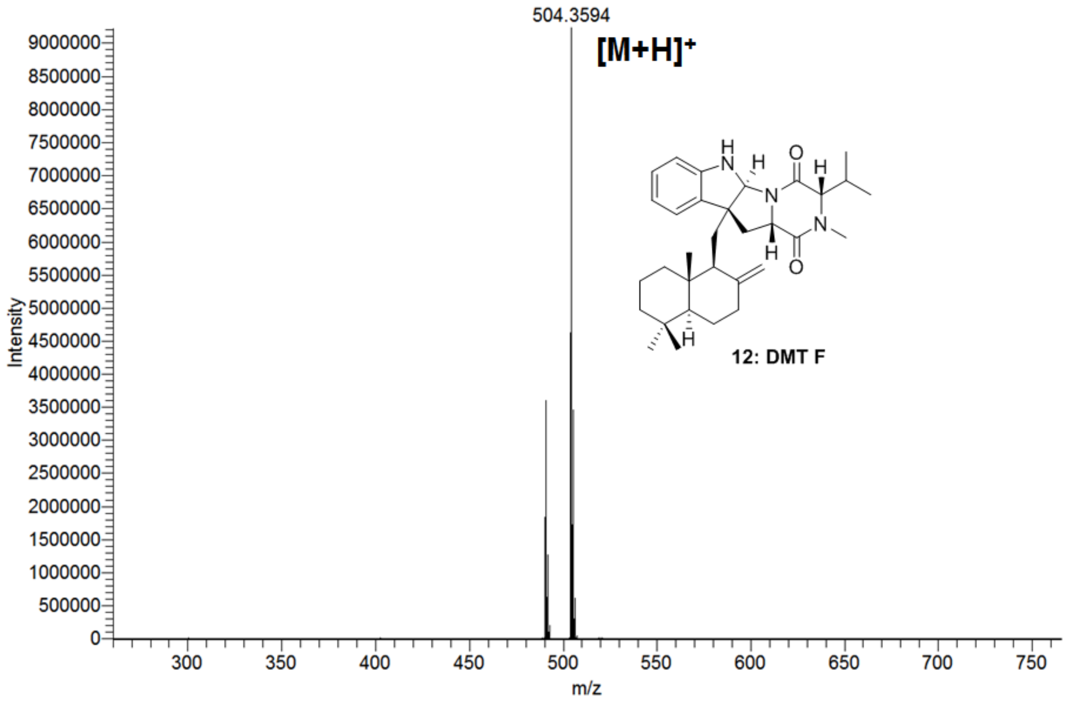

Supplementary Figure 26. HR-ESIMS spectrum of compound **12** (DMT F).

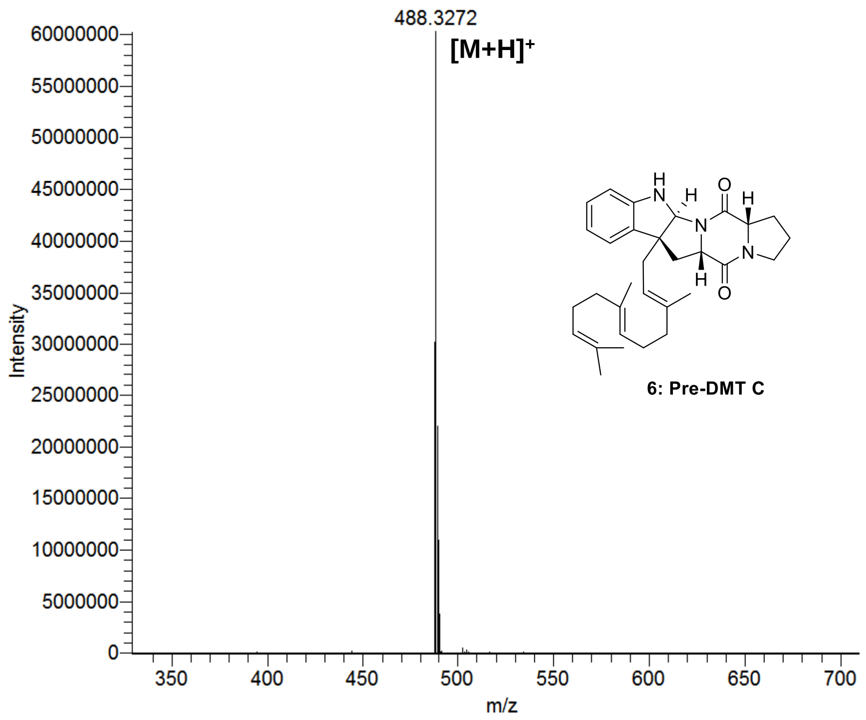

Supplementary Figure 27. HR-ESIMS spectrum of compound **6** (pre-drimentine C)**.**

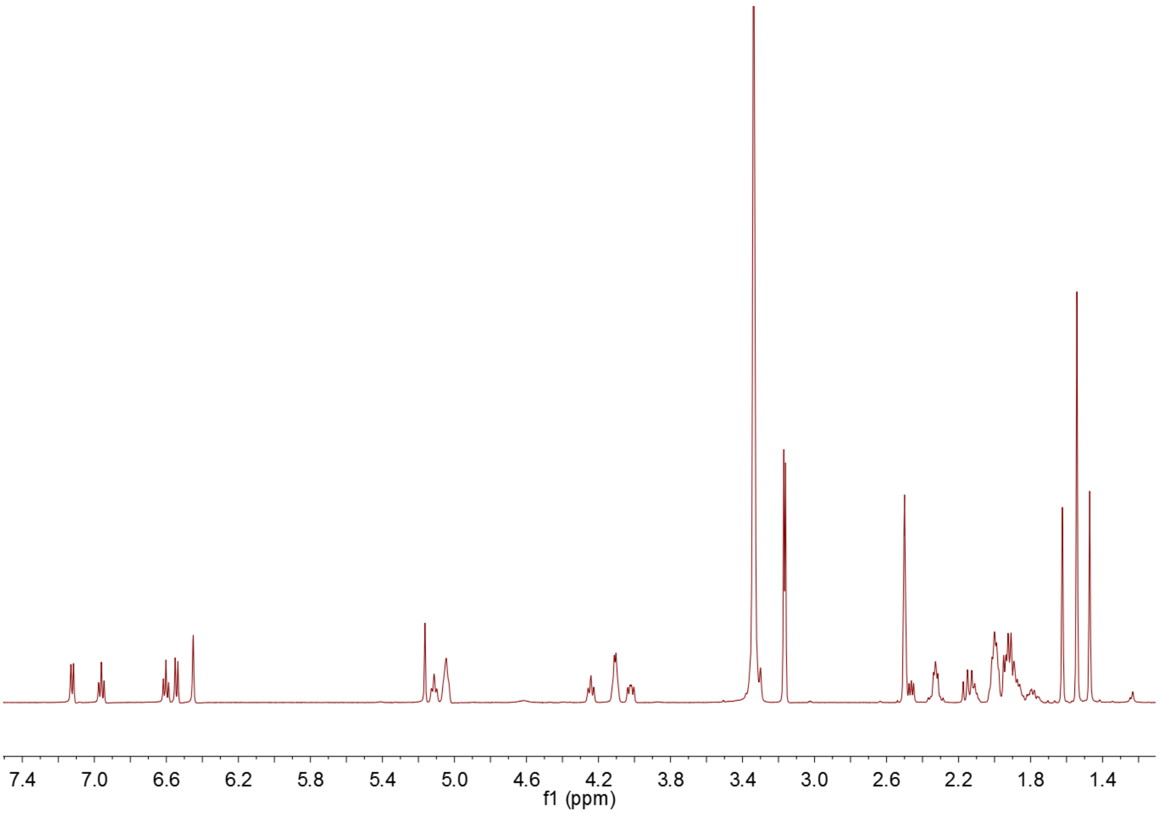

Supplementary Figure 28. ^{1}H NMR spectrum of compound **6** in DMSO-d_6.

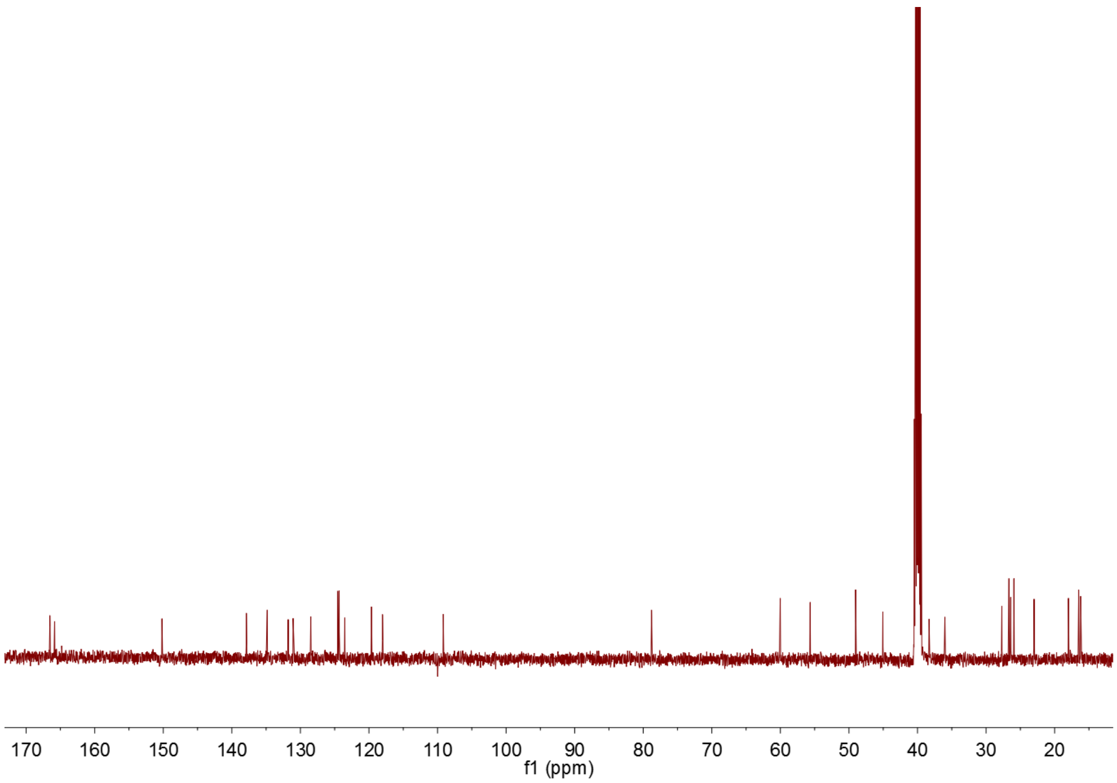

Supplementary Figure 29. ^{13}C NMR spectrum of compound **6** in DMSO-d_6.

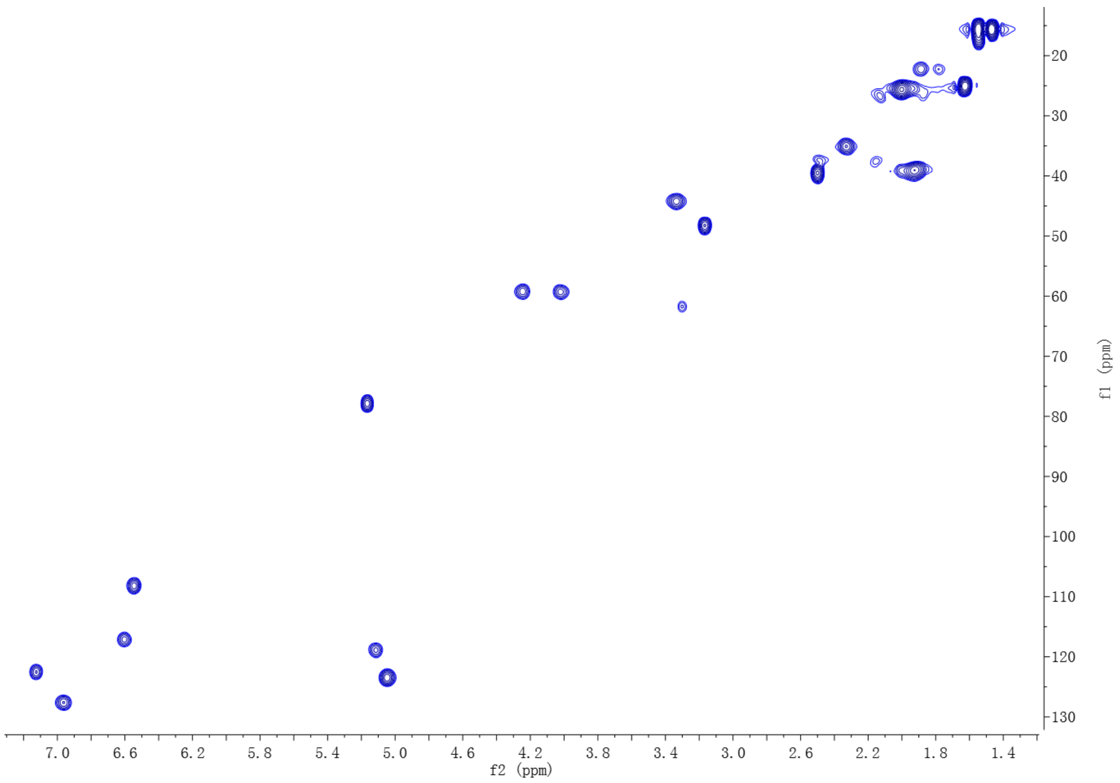

Supplementary Figure 30. HSQC spectrum of compound **6** in DMSO-d_6.

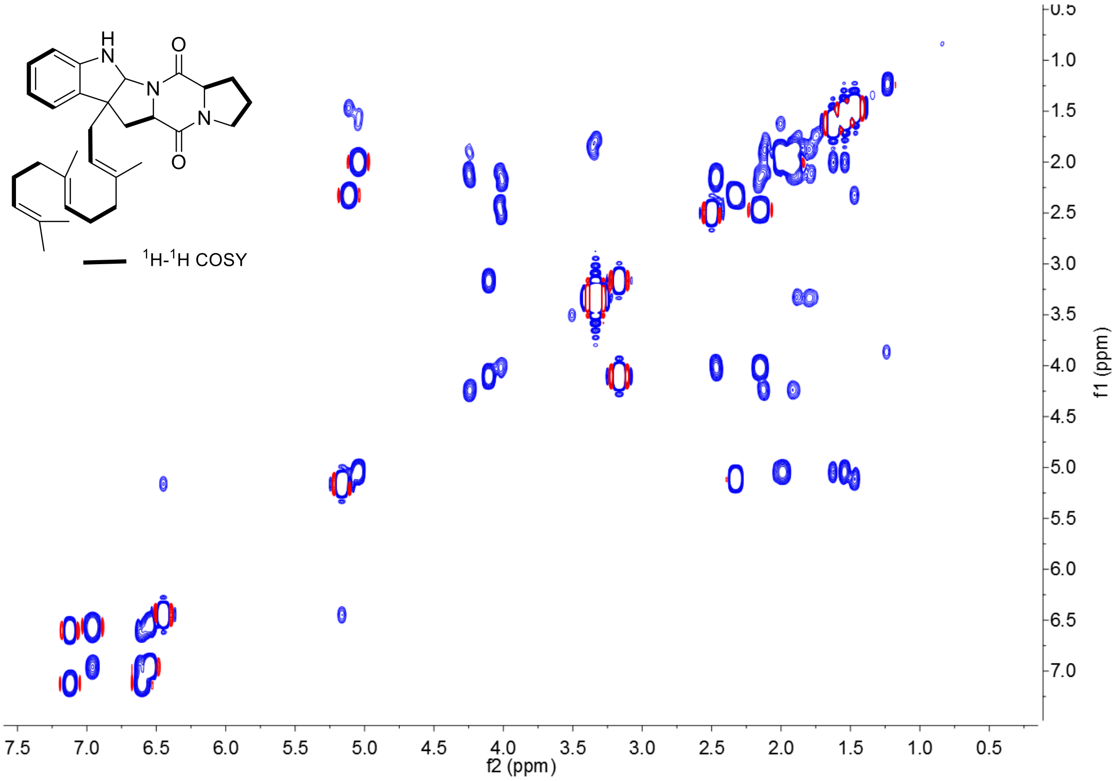

Supplementary Figure 31. COSY spectrum of compound **6** in DMSO-d_6.

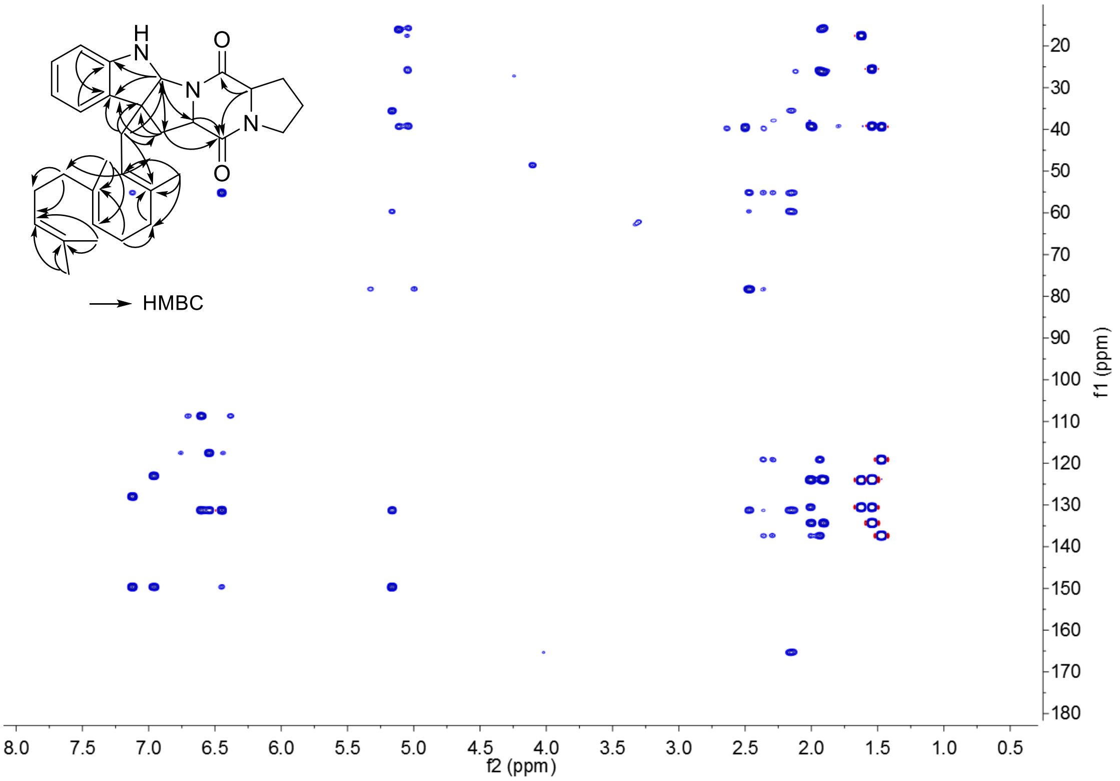

Supplementary Figure 32. HMBC spectrum of compound **6** in DMSO-d_6.

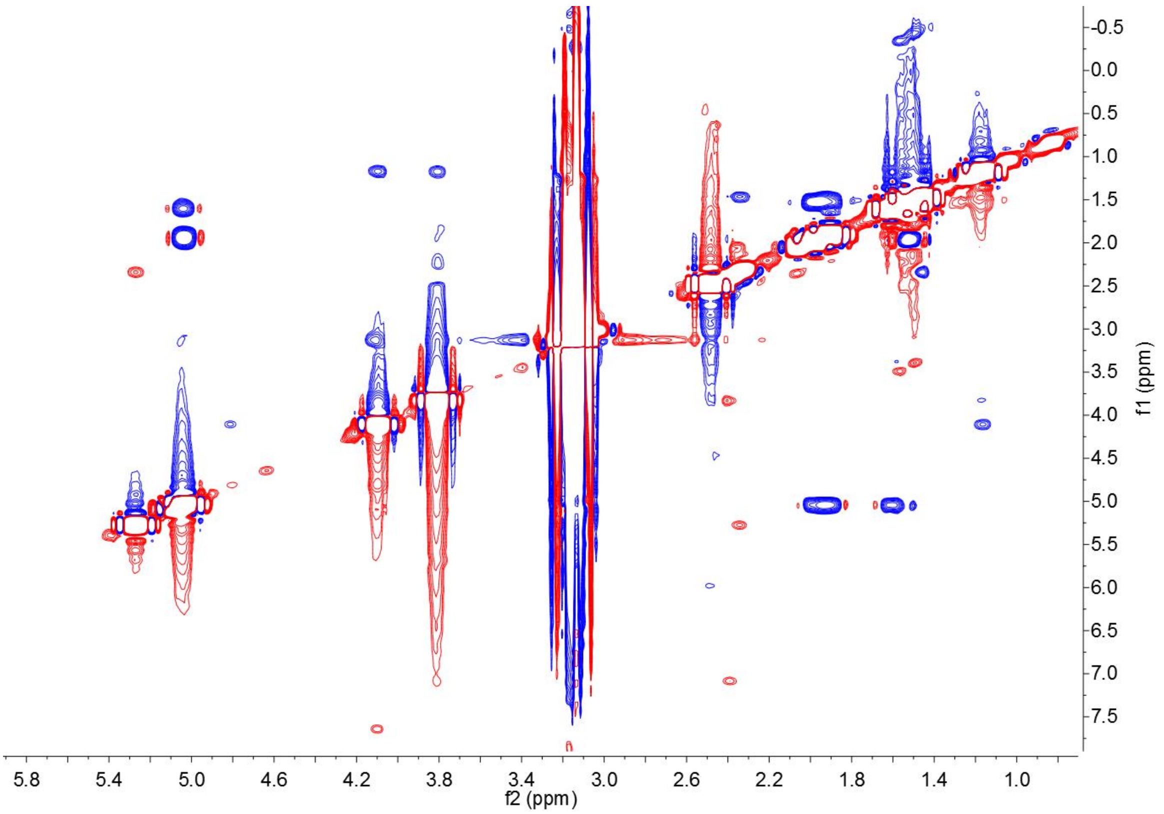

Supplementary Figure 33. NOESY spectrum of compound **6** in DMSO-d_6.

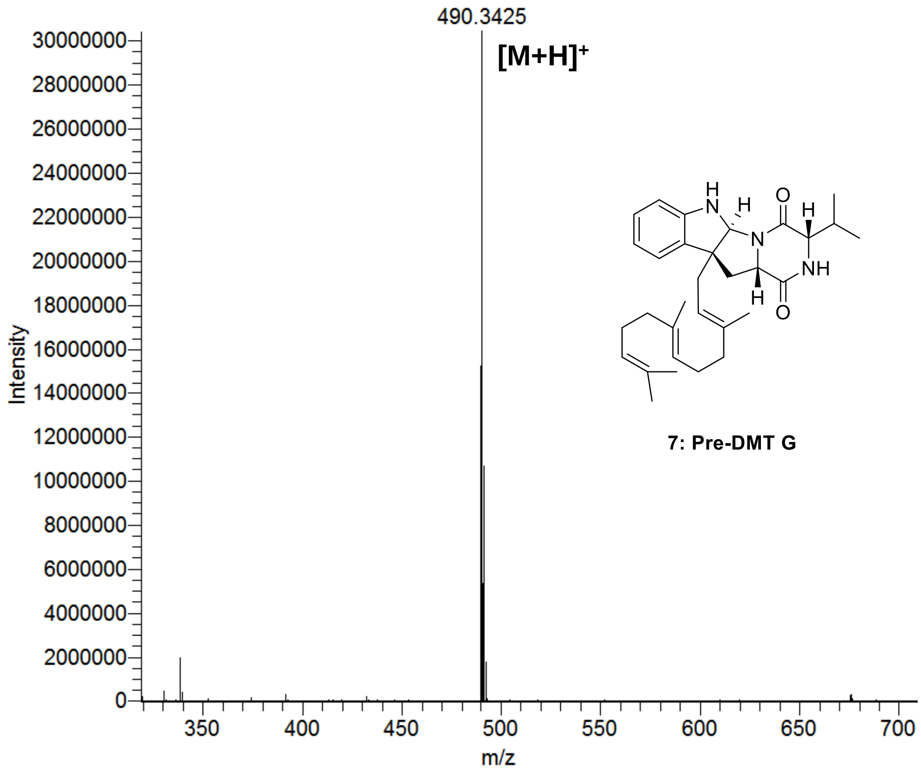

Supplementary Figure 34. HR-ESIMS spectrum of compound **7** (pre-drimentine G)**.**

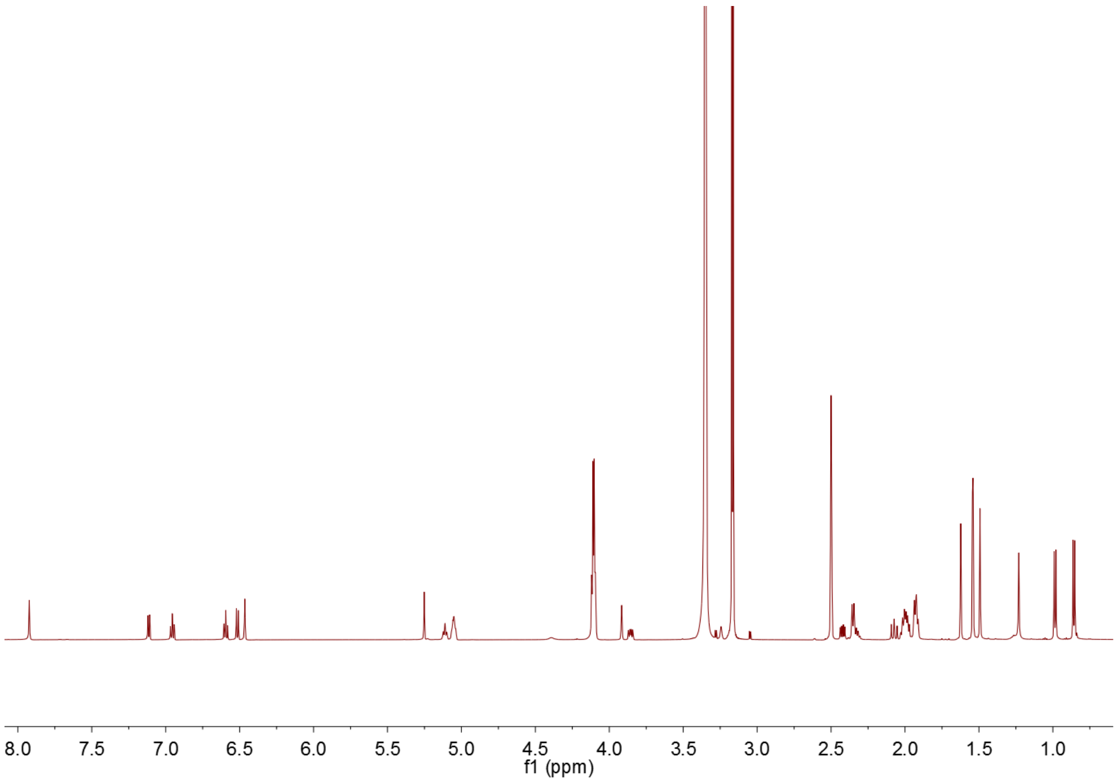

Supplementary Figure 35. ^{1}H NMR spectrum of compound **7** in DMSO-d_6.

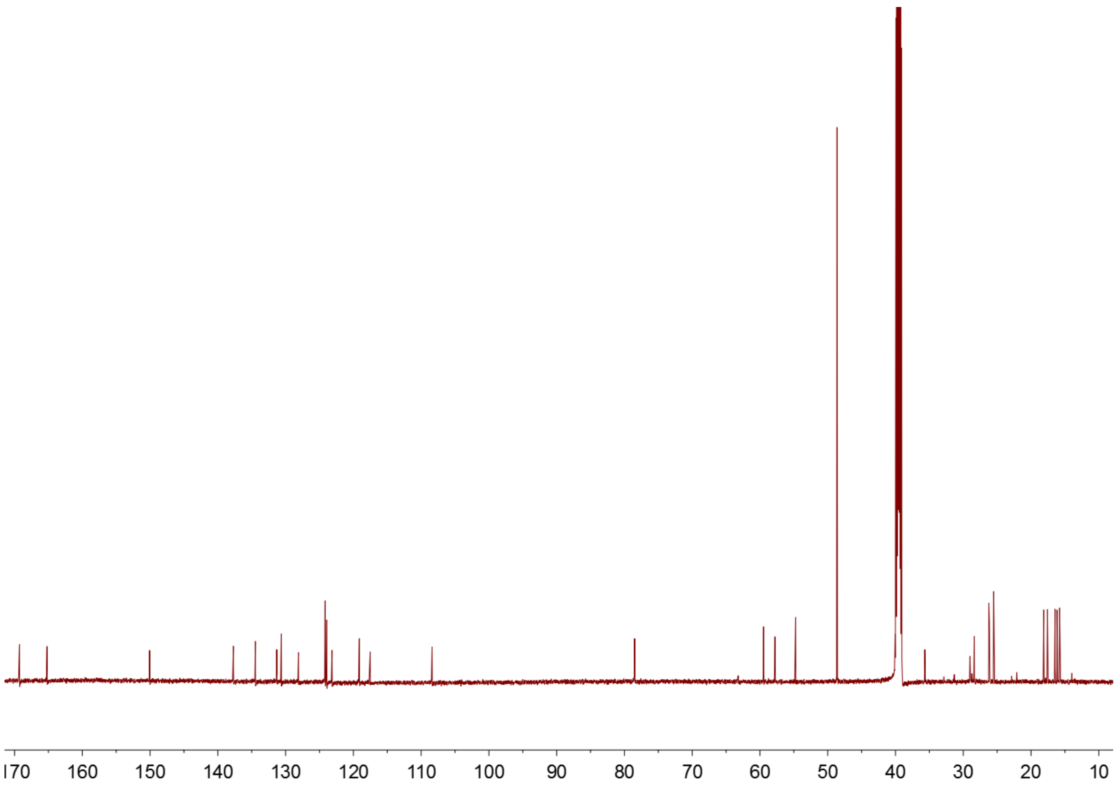

Supplementary Figure 36. ^{13}C NMR spectrum of compound **7** in DMSO-d_6.

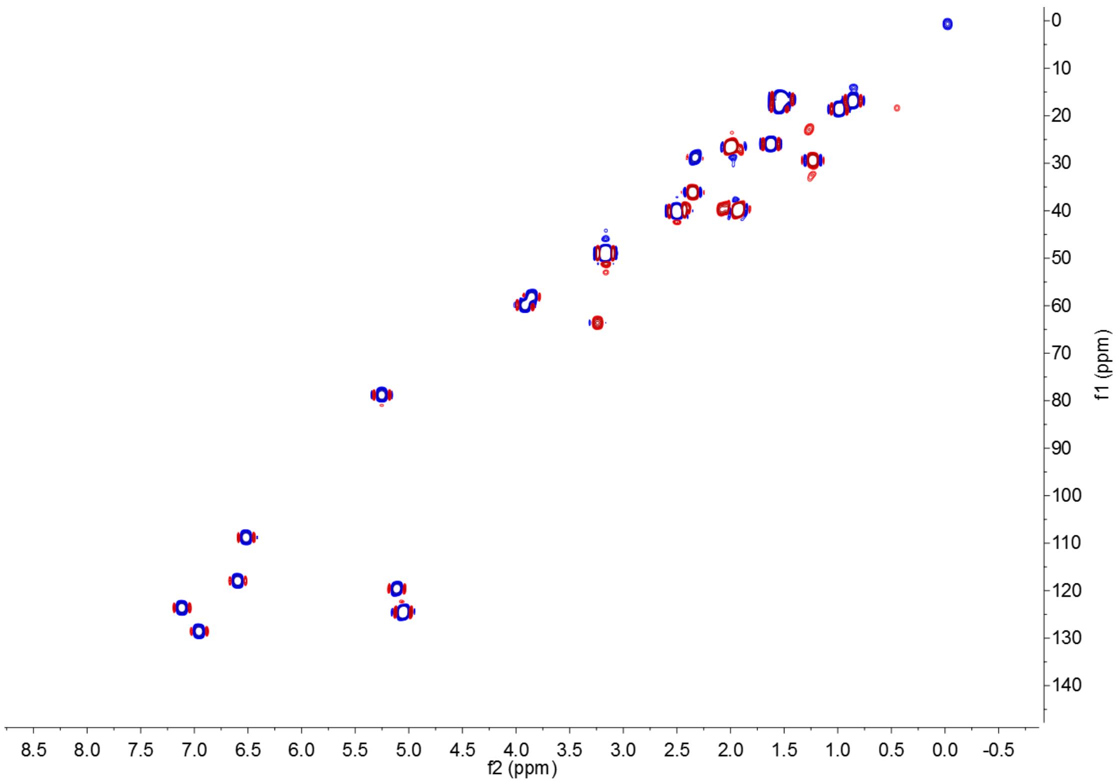

Supplementary Figure 37. HSQC spectrum of compound **7** in DMSO-d_6.

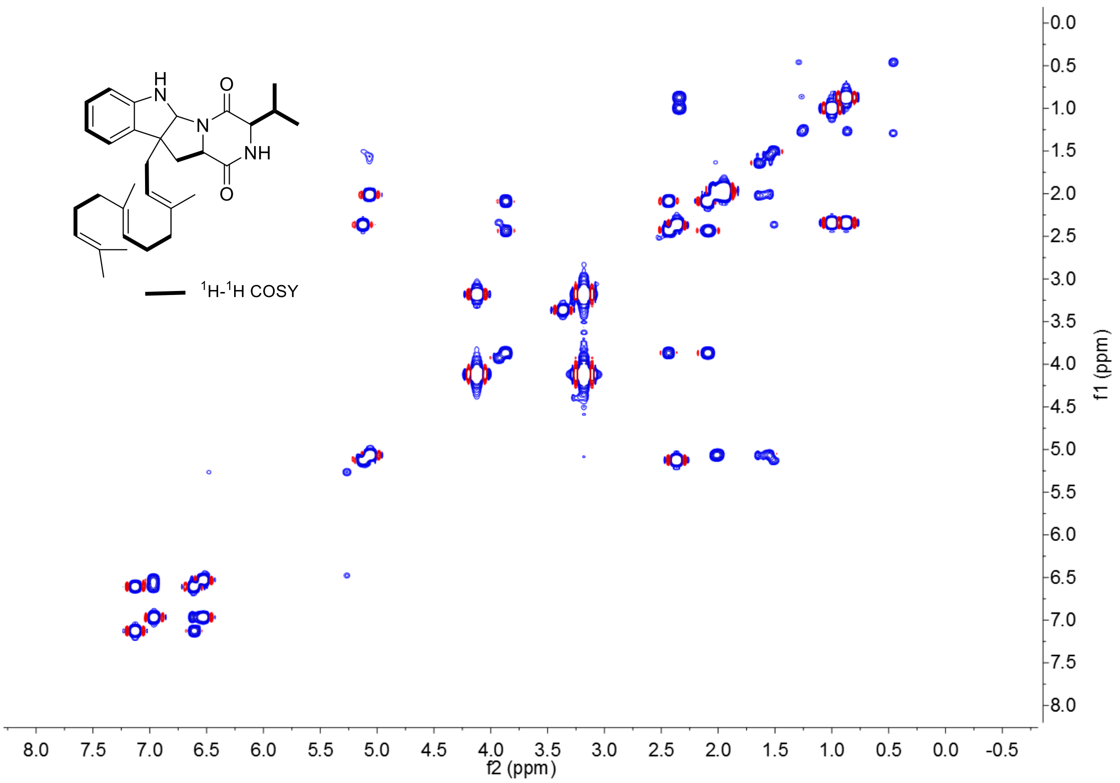

Supplementary Figure 38. COSY spectrum of compound **7** in DMSO-d_6.

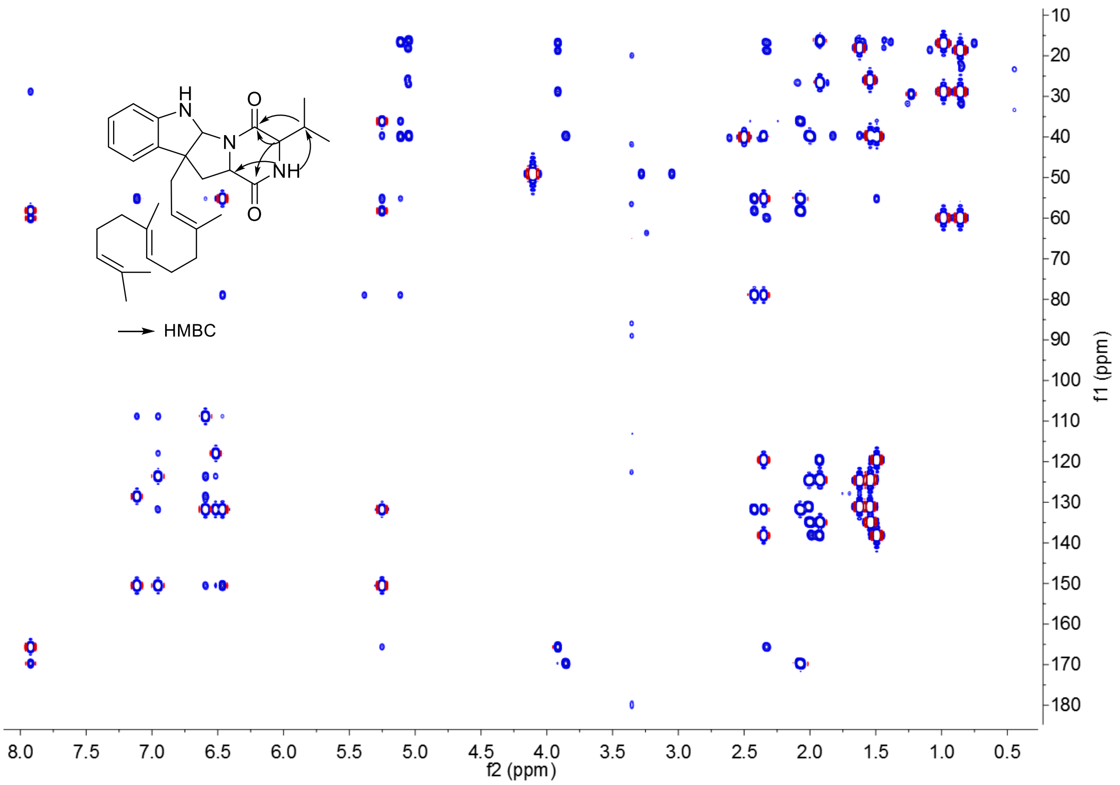

Supplementary Figure 39. HMBC spectrum of compound **7** in DMSO-d_6.

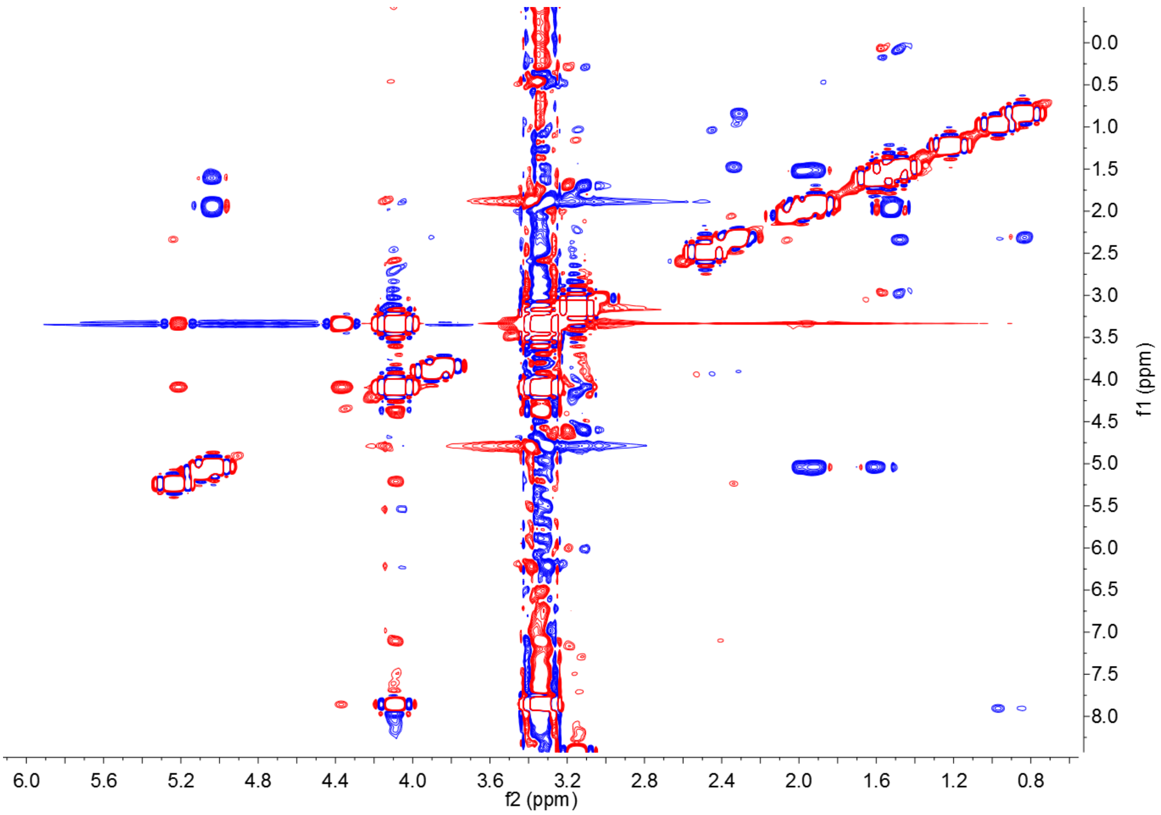

Supplementary Figure 40. NOESY spectrum of compound **7** in DMSO-d_6.

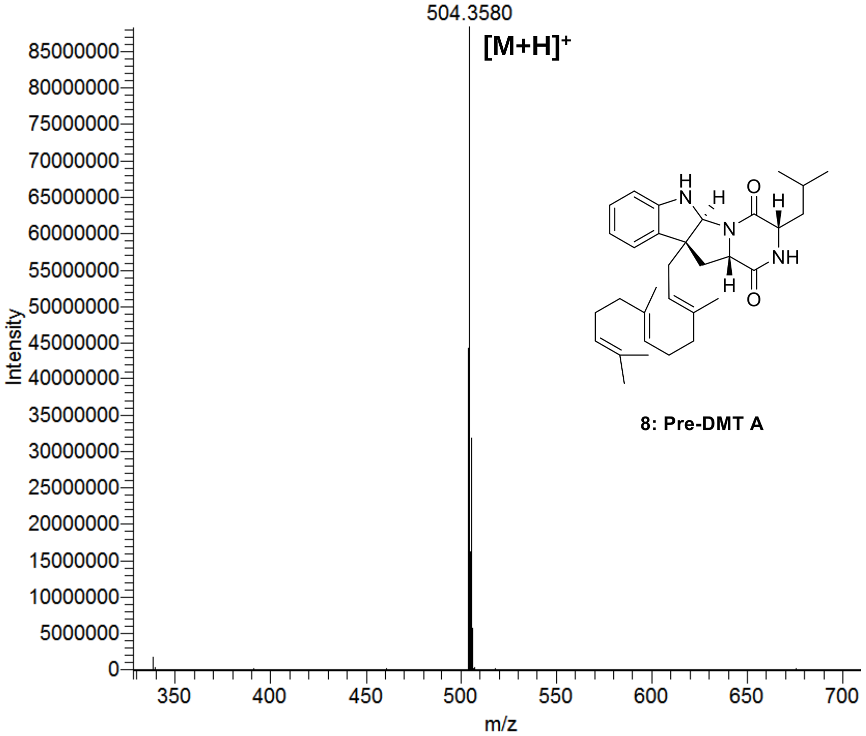

Supplementary Figure 41. HR-ESIMS spectrum of compound **8** (pre-drimentine A)**.**

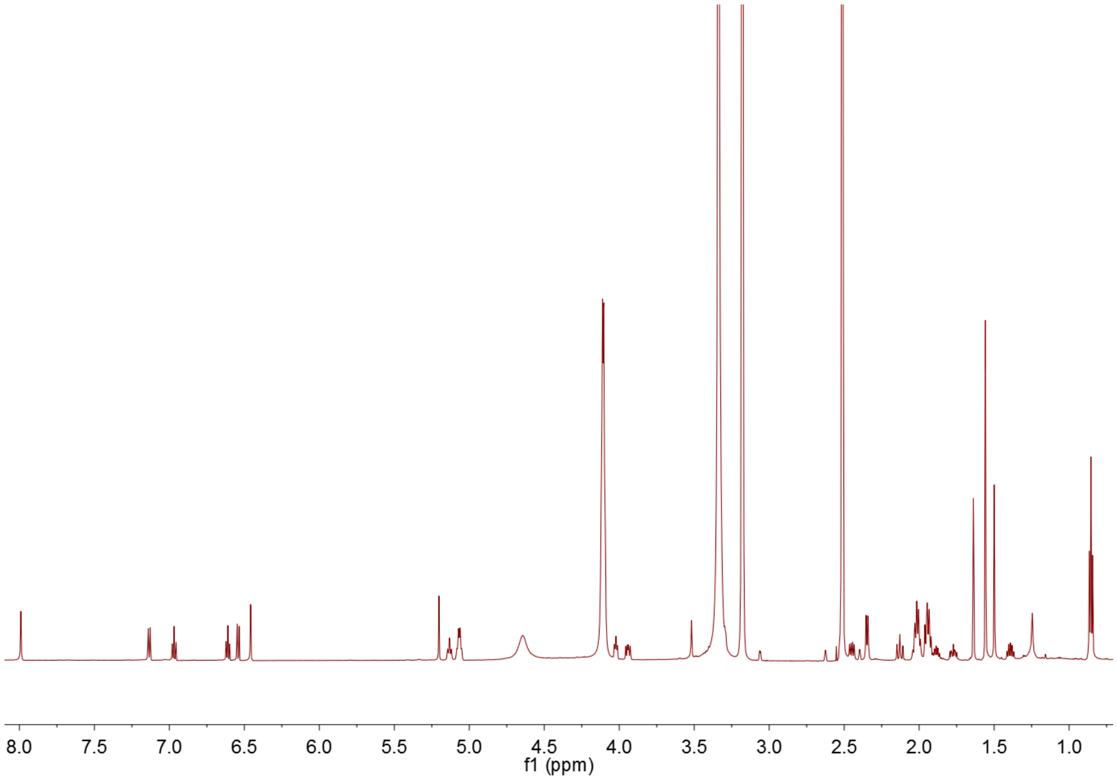

Supplementary Figure 42. ^{1}H NMR spectrum of compound **8** in DMSO-d_6.

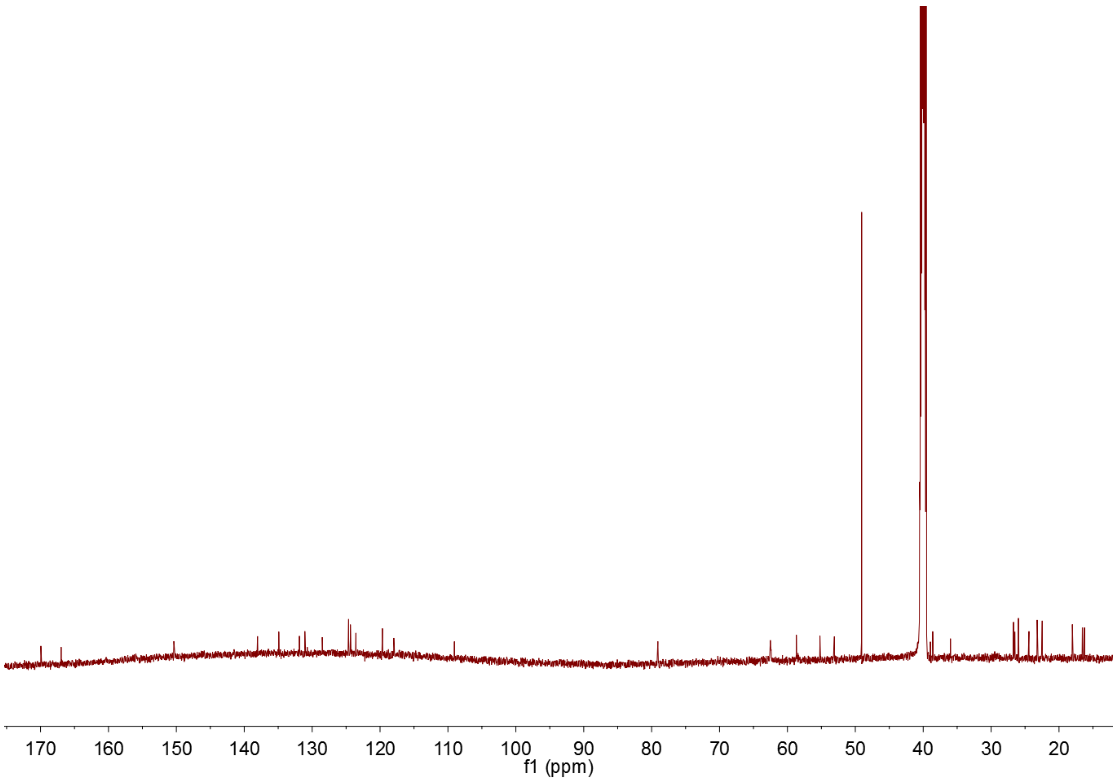

Supplementary Figure 43. ^{13}C NMR spectrum of compound **8** in DMSO-d_6.

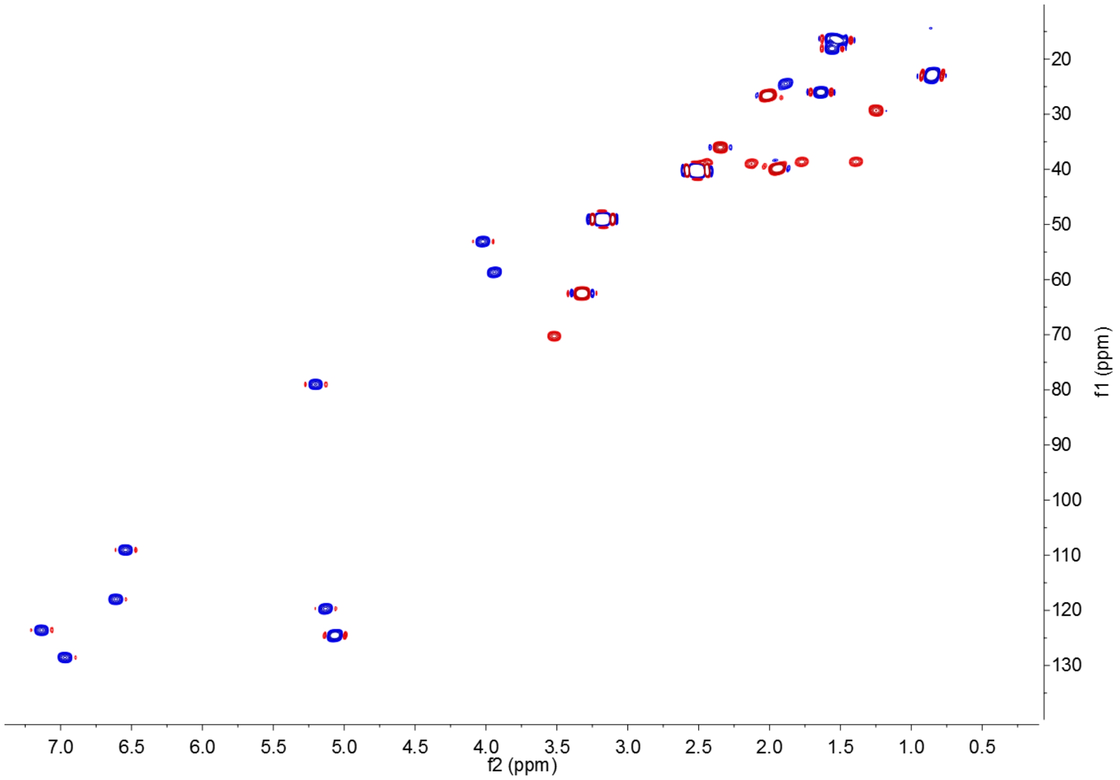

Supplementary Figure 44. HSQC spectrum of compound **8** in DMSO-d_6.

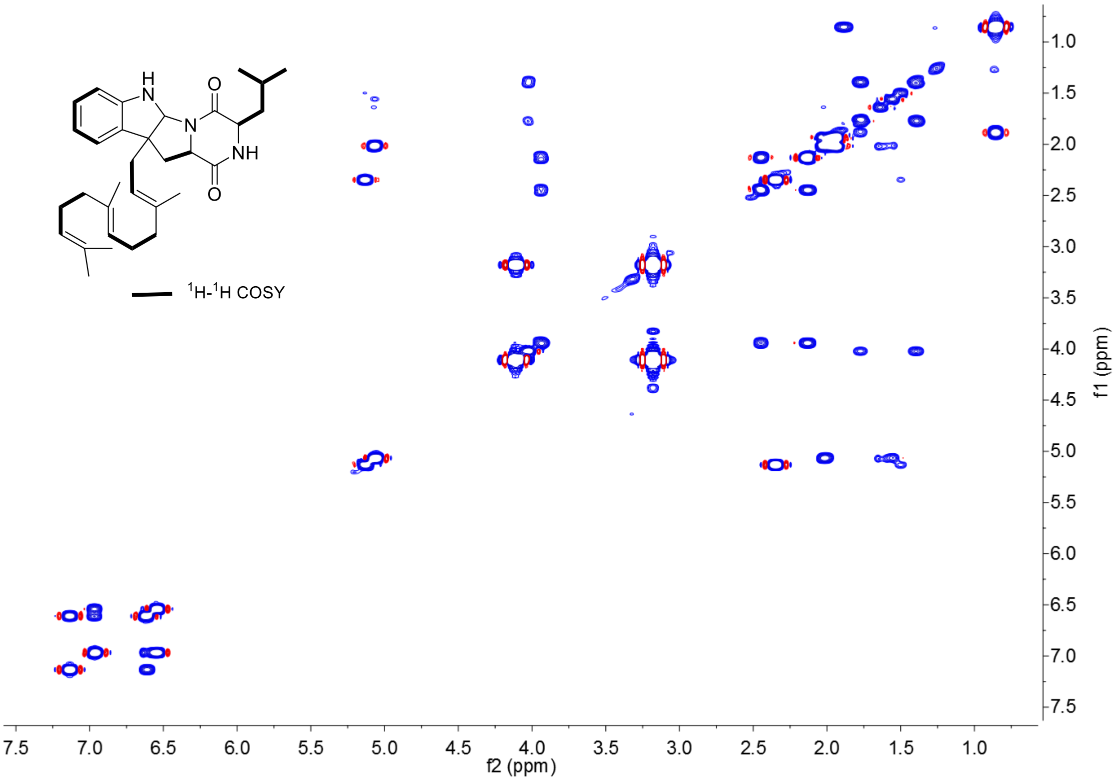

Supplementary Figure 45. COSY spectrum of compound **8** in DMSO-d_6.

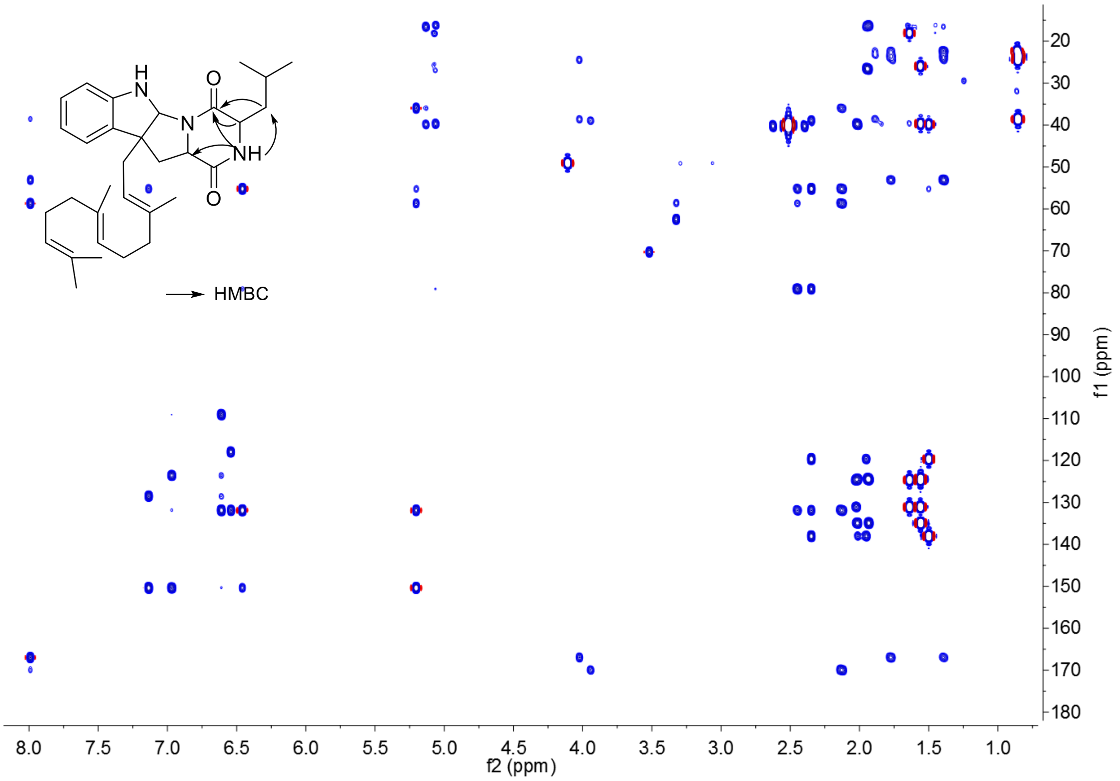

Supplementary Figure 46. HMBC spectrum of compound **8** in DMSO-d_6.

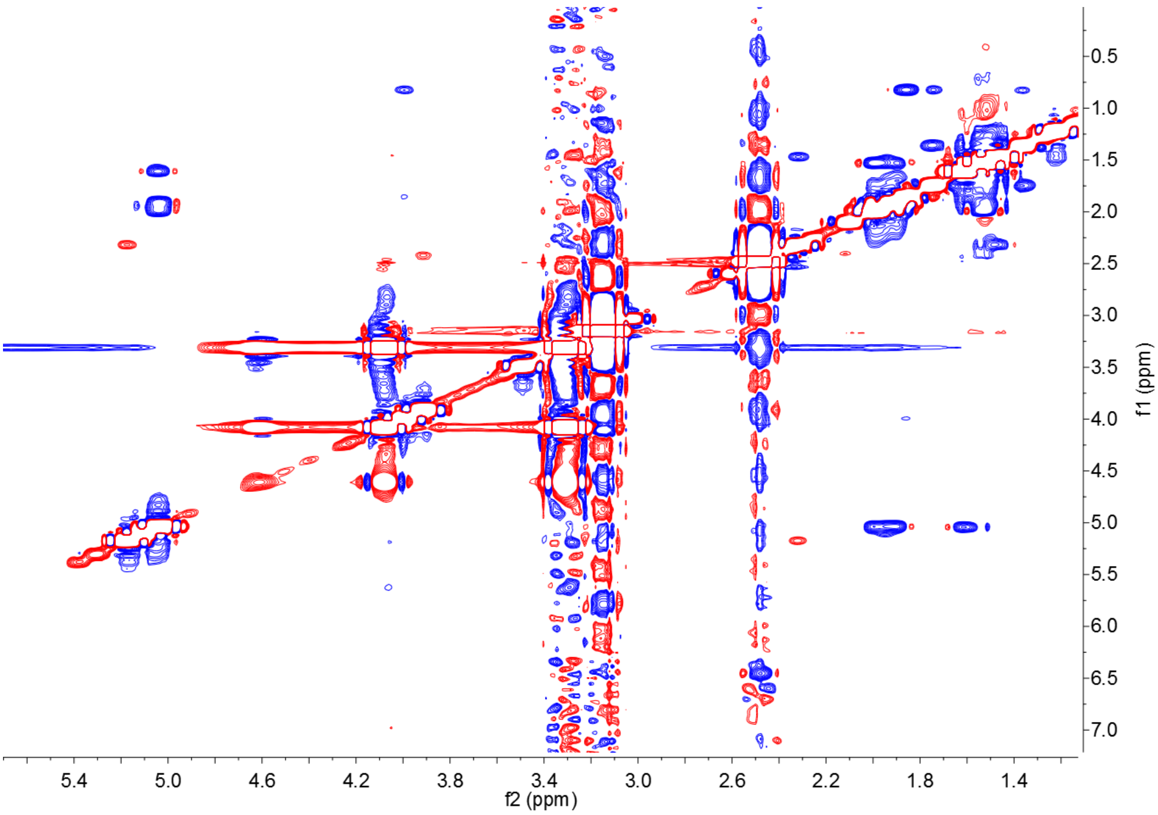

Supplementary Figure 47. NOESY spectrum of compound **8** in DMSO-d_6.

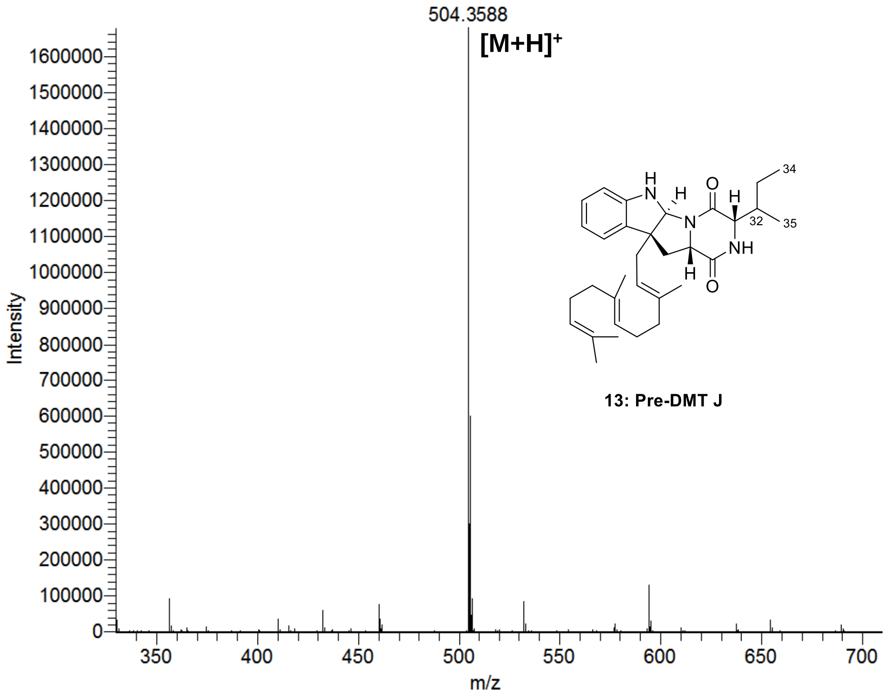

Supplementary Figure 48. HR-ESIMS spectrum of compound **13** (pre-drimentine J).

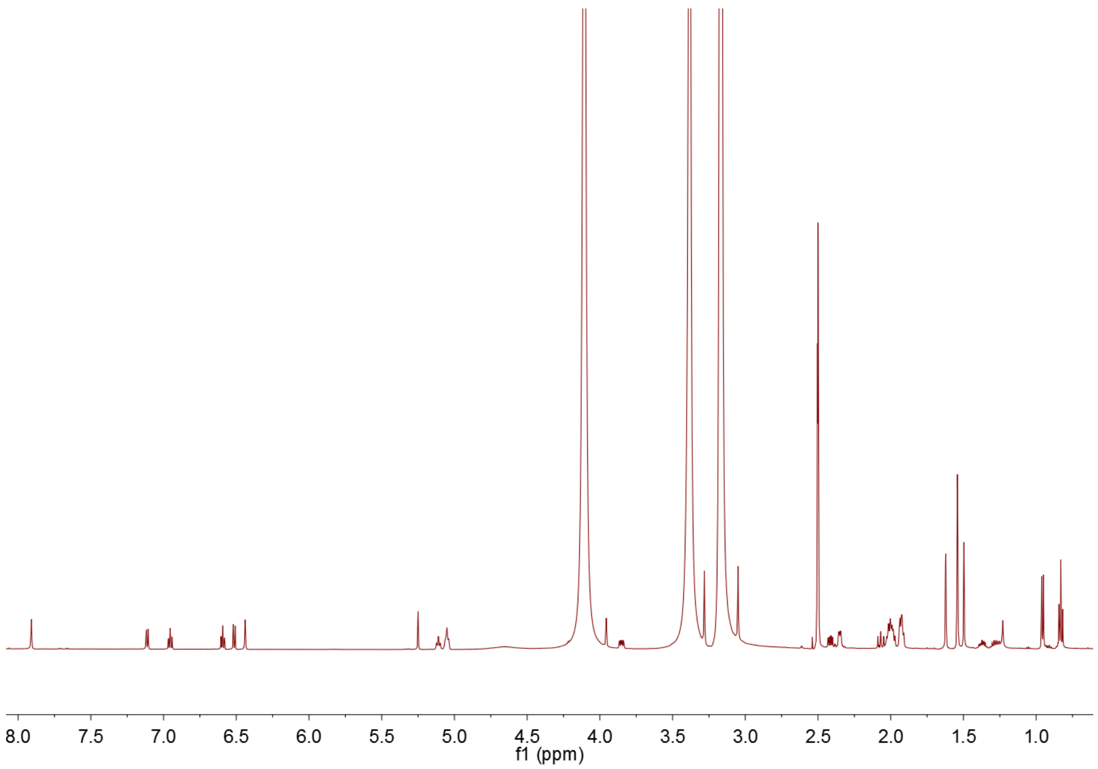

Supplementary Figure 49. ^{1}H NMR spectrum of compound **13** in DMSO-d_6.

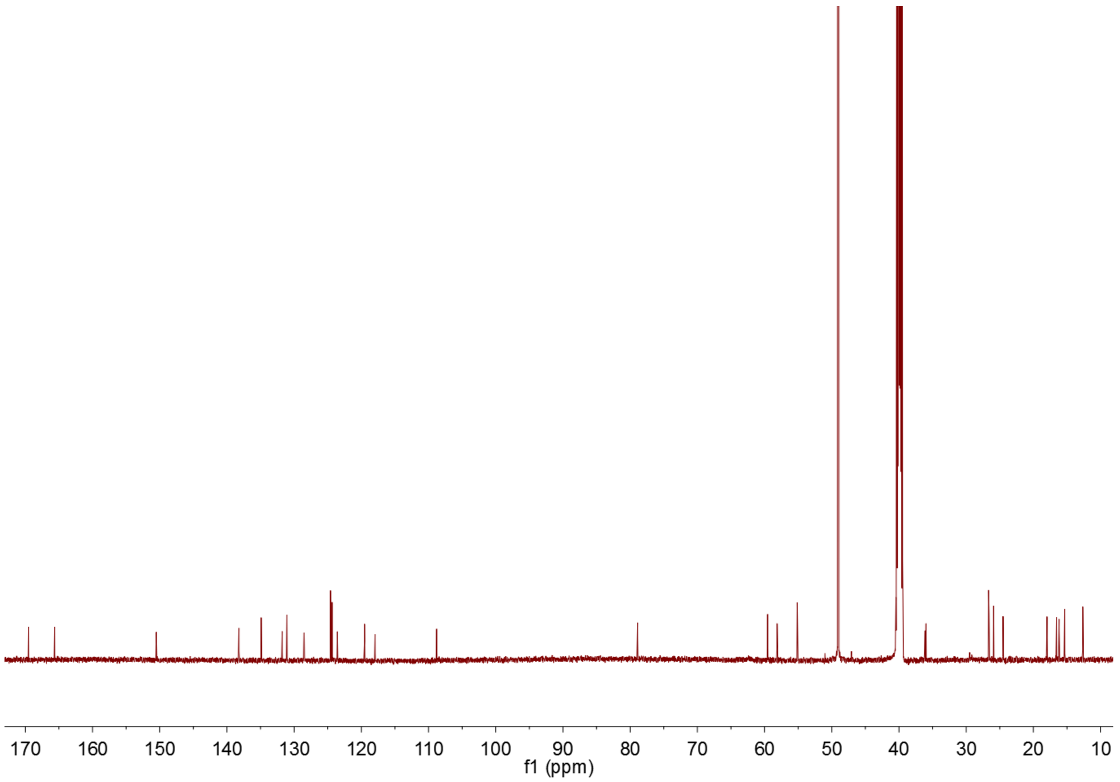

Supplementary Figure 50. ^{13}C NMR spectrum of compound **13** in DMSO-d_6.

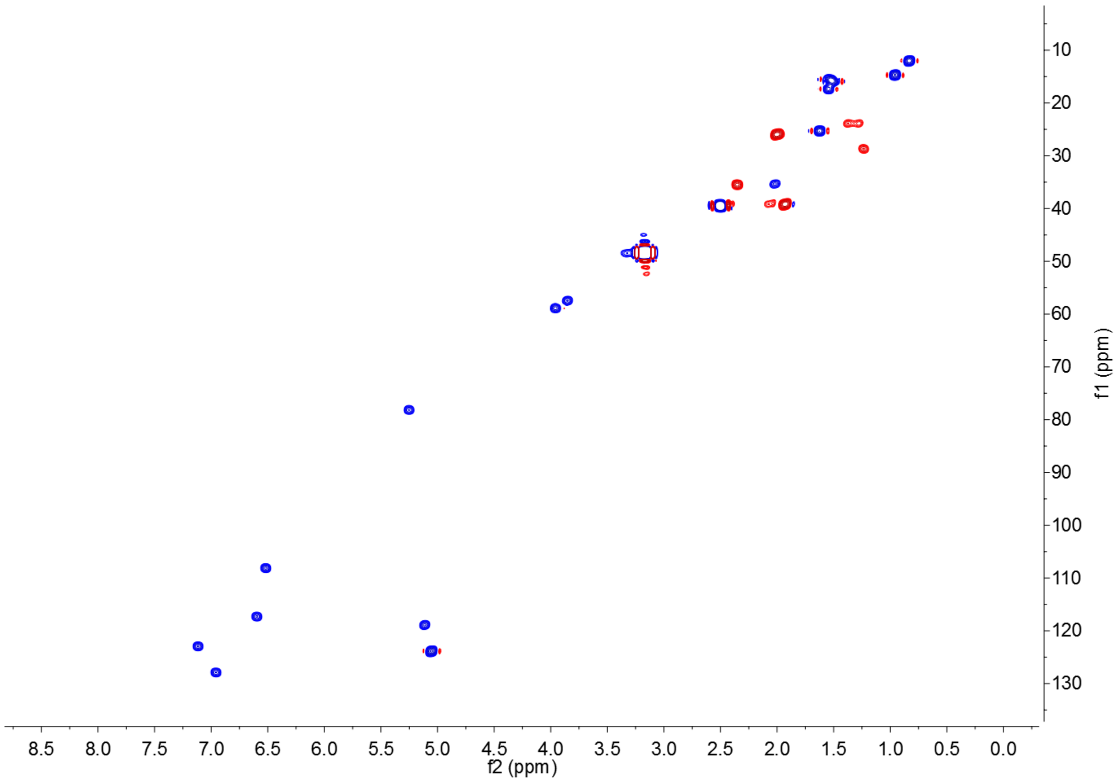

Supplementary Figure 51. HSQC spectrum of compound **13** in DMSO-d_6.

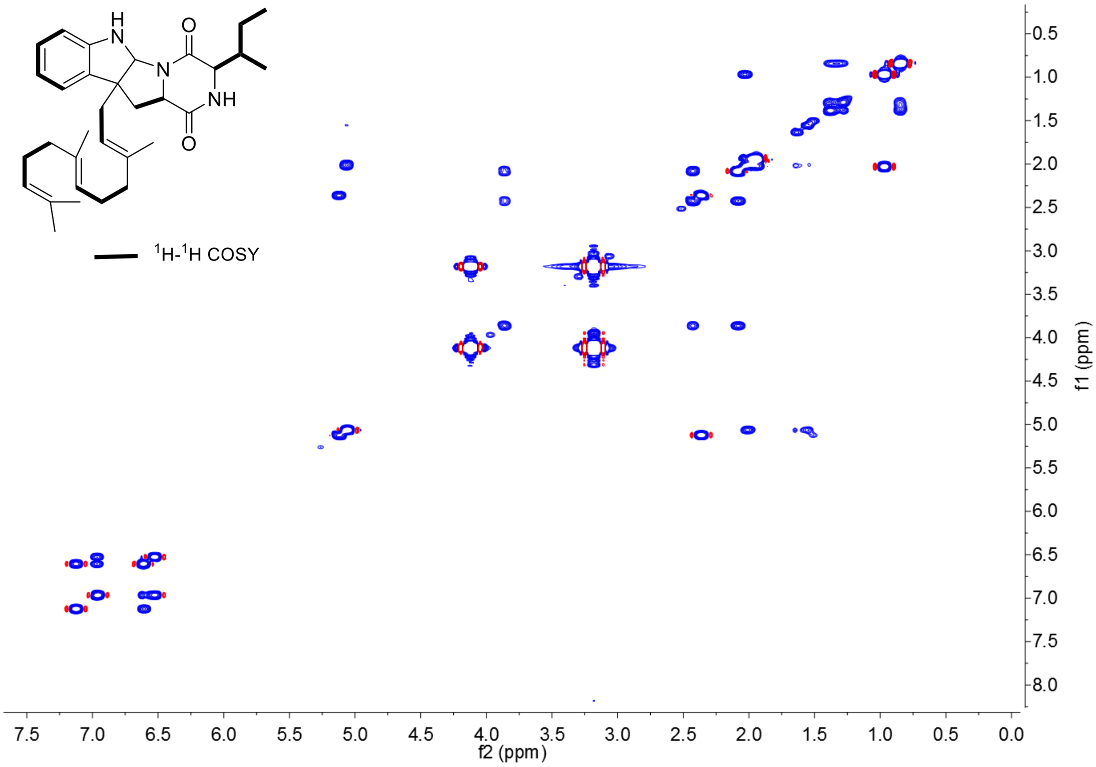

Supplementary Figure 52. COSY spectrum of compound **13** in DMSO-d_6.

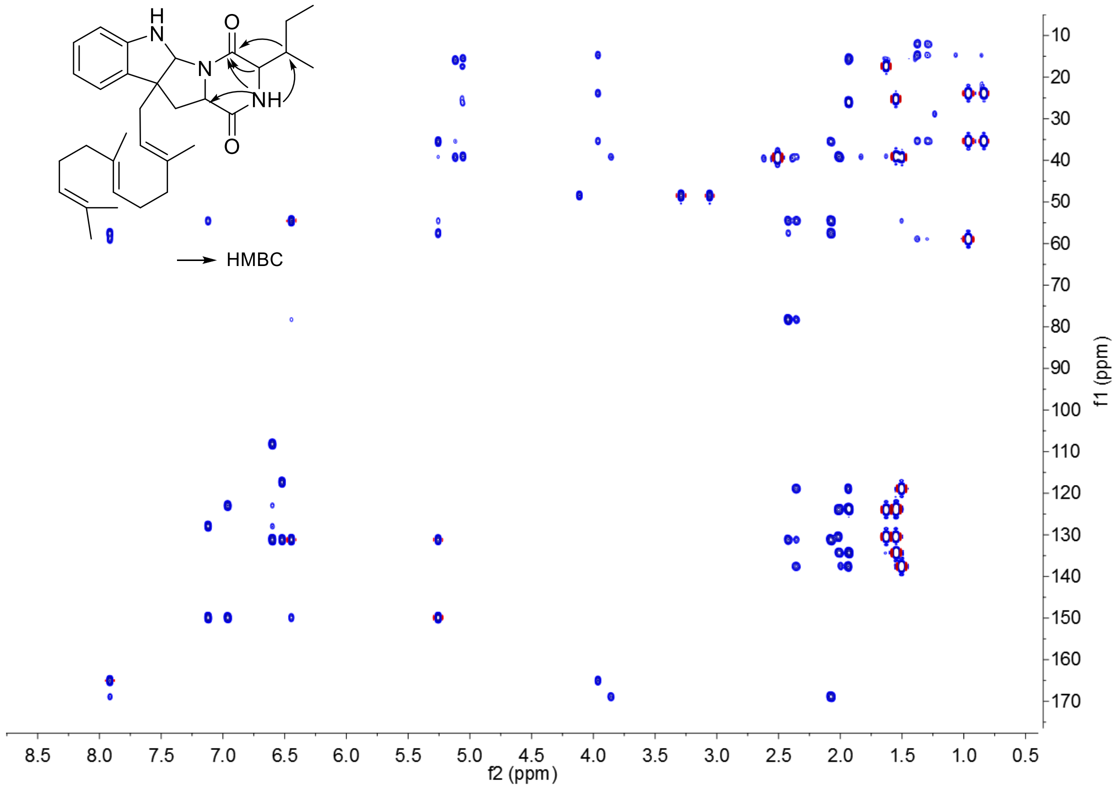

Supplementary Figure 53. HMBC spectrum of compound **13** in DMSO-d_6.

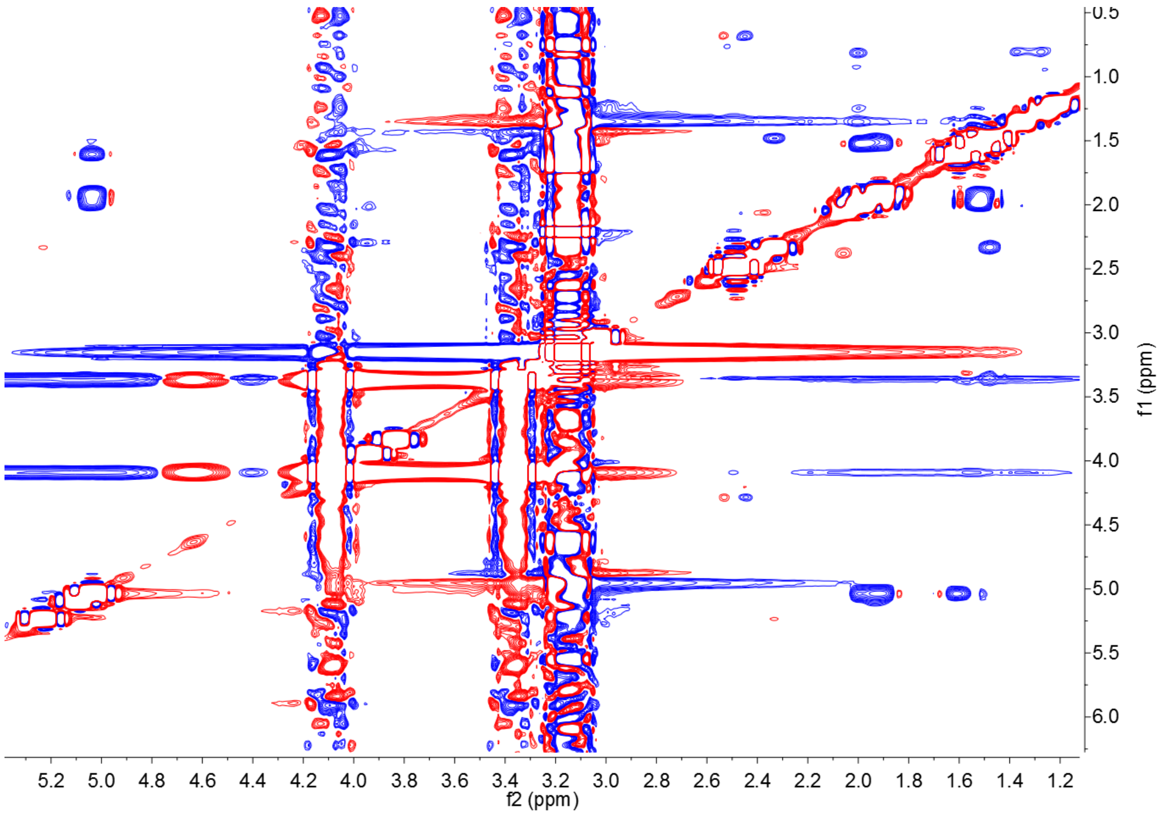

Supplementary Figure 54. NOESY spectrum of compound **13** in DMSO-d_6.

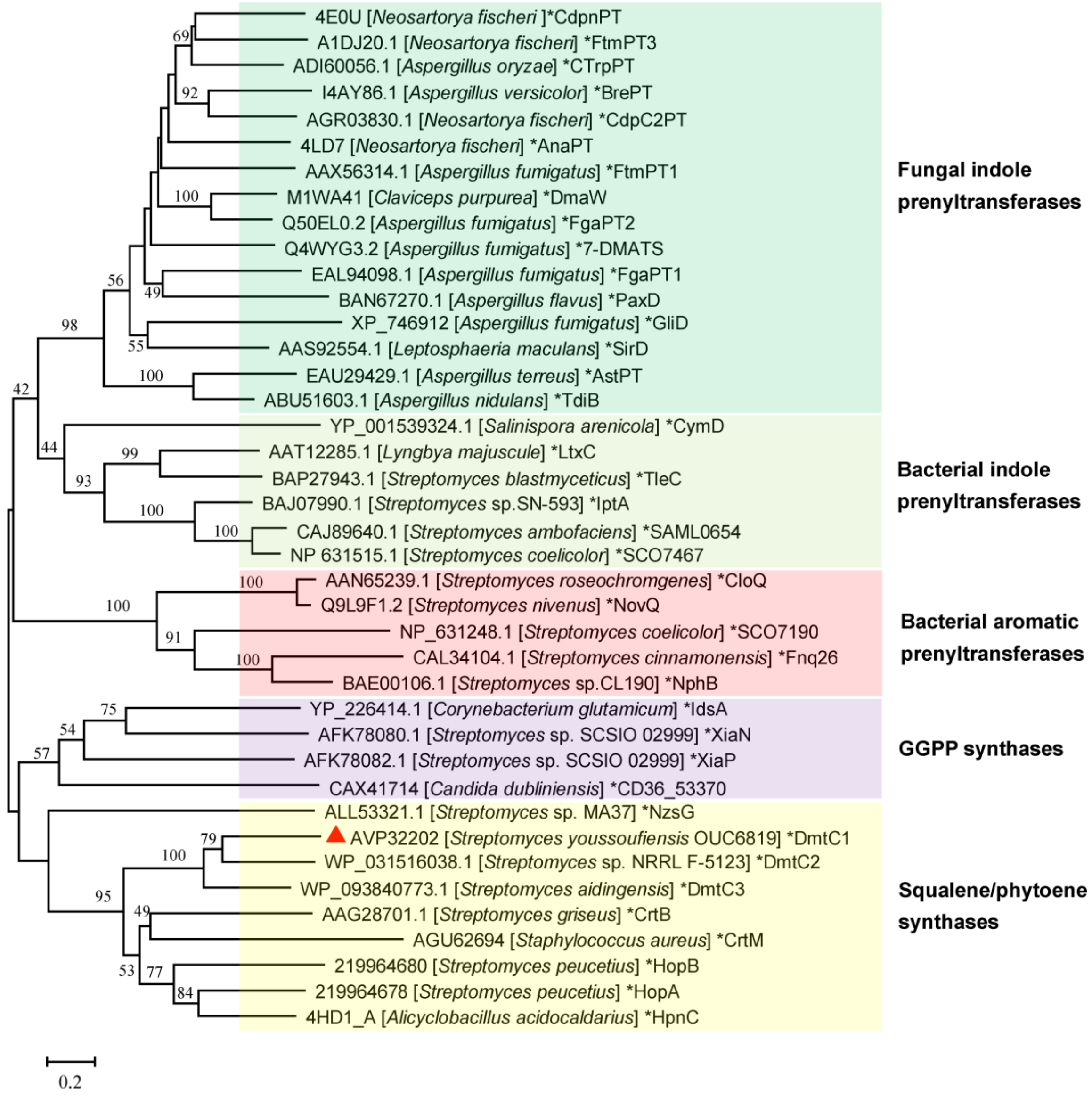

Supplementary Figure 55. Phylogenetic analysis of DmtC1-3 with selected functionally characterized PTs. A phylogenetic tree was generated in MEGA 6[7] by using neighbor-joining method[8] with 1,000 bootstrapping cycles. Numbers at each branch point indicate the bootstrap values. The accession numbers of sequences as well as their origins are shown. Scale bar represents 20% replacement of amino acids per site.

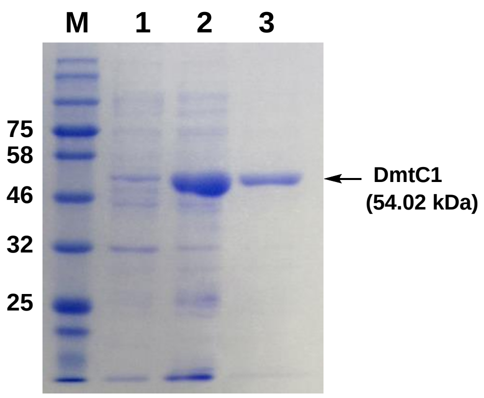

Supplementary Figure 56. SDS-PAGE (12%) analysis of expression and purification of DmtC1. Lane M, protein marker; lane 1, supernatant of *E.coli* BL21 (DE3) /pET32a; lane 2, supernatant of *E.coli* BL21 (DE3) /pET32a::*dmtC1*; lane 3, purified recombinant DmtC1 with a calculated molecular weight of 54.02 kDa.

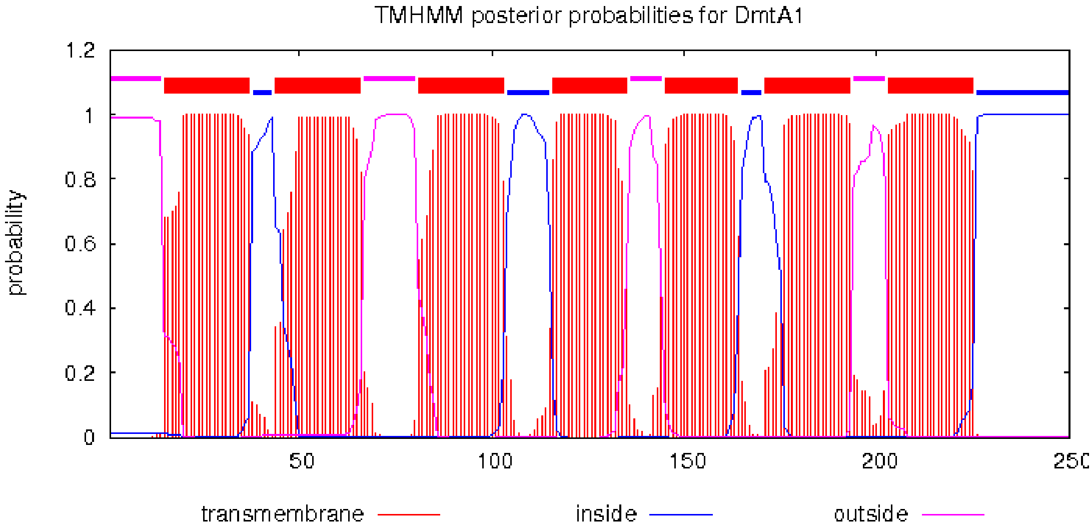

Supplementary Figure 57. Prediction of transmembrane helices for DmtA1 by TMHMM Server v. 2.0. DmtA1 is predicted to contain seven transmembrane helices. X-axis represents the residue number, while Y-axis shows the probability of orientation. Red lines indicate transmembrane helices, blue lines indicate intervening loop regions located inside, and fuchsia lines represent intervening loop regions located outside.

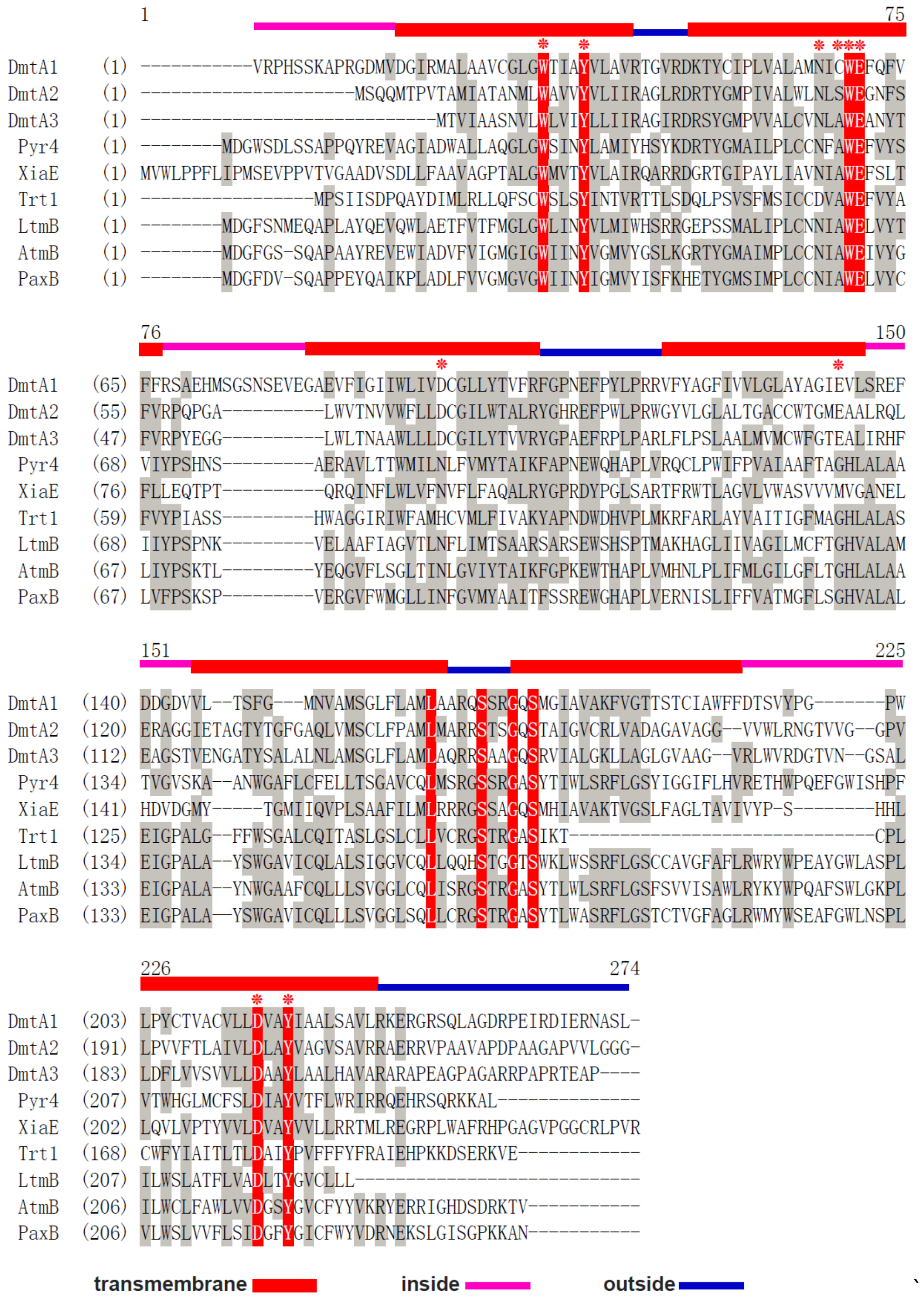

Supplementary Figure 58. Multiple-sequence alignment of DmtA1-3 with other membrane TCs. The origins of the TCs are as follows: DmtA1 (AVP32200) from *S. youssoufiensis* OUC6819, DmtA2 (WP_052397357.1) from *Streptomyces* sp. NRRL F-5123, DmtA3 (SFD40844.1) from *S. aidingensis* CGMCC 4.5739, XiaE (CCH63731.1) from *Streptomyces* sp. HKI0576, Pyr4 (XP_751270.1) from *Aspergillus fumigatus*, Trt1 (ATEG_10077) from *Aspergillus terreus*, AtmB (AM921700) from *Aspergillus flavus*, PaxB (AF279808) from *Penicillium paxilli*, and LtmB (DQ443465) from *Neotyphodium lolii*. Predicted protein secondary structure of DmtA1 is shown above the sequences. Conserved residues are colored in red background. Amino acids subjected to mutagenesis in this study are indicated by red asterisks.

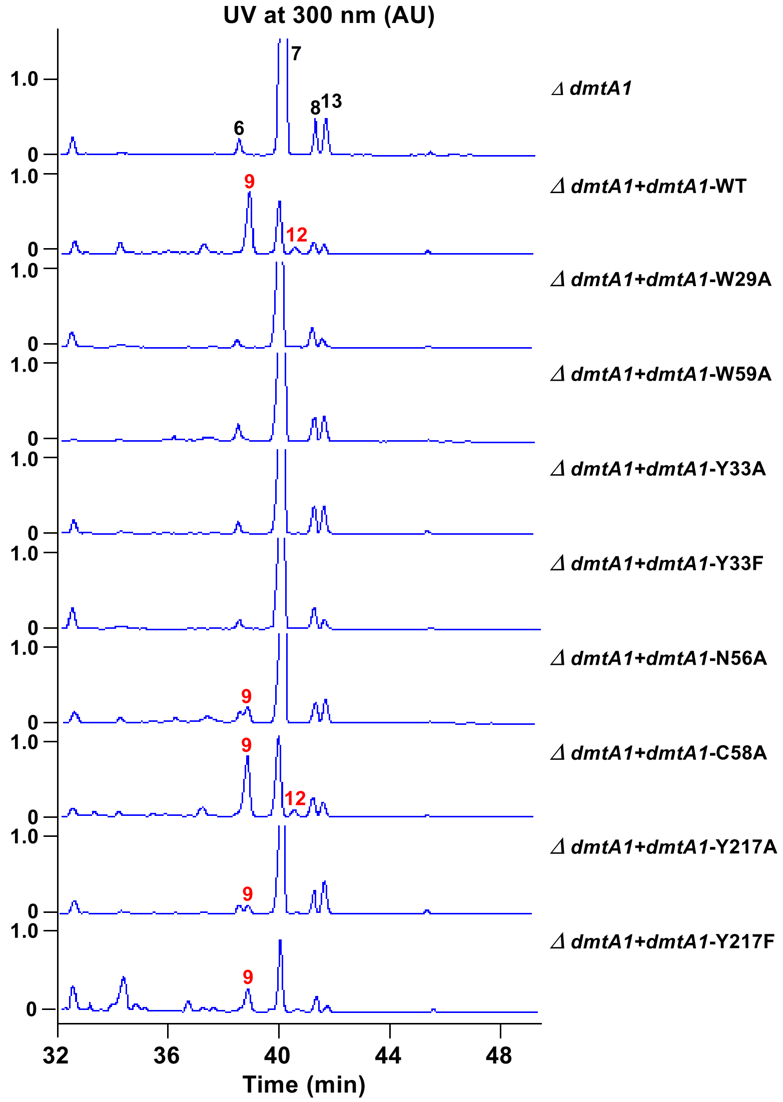

Supplementary Figure 59. HPLC traces of Δ*dmtA1* complemented with different *dmtA1* variants. The cyclization products **9** and **12** were indicated in red.

Supplementary References

1 Guindon, S. *et al.* New algorithms and methods to estimate maximum-likelihood phylogenies: assessing the performance of PhyML 3.0. *Syst. Biol.* **59**, 307-321 (2010).

2 Gouy, M., Guindon, S. & Gascuel, O. SeaView version 4: a multiplatform graphical user interface for sequence alignment and phylogenetic tree building. *Mol. Biol. Evol.* **27**, 221-224 (2009).

3 Letunic, I. & Bork, P. Interactive tree of life (iTOL) v3: an online tool for the display and annotation of phylogenetic and other trees. *Nucleic Acids Res.* **44**, W242-W245 (2016).

4 Bonnefond, L. *et al.* Structural basis for nonribosomal peptide synthesis by an aminoacyl-tRNA synthetase paralog. *Proc Natl Acad Sci U S A* **108**, 3912-3917 (2011).

5 Roy, A., Kucukural, A. & Zhang, Y. I-TASSER: a unified platform for automated protein structure and function prediction. *Nat. Protoc.* **5**, 725-738 (2010).

6 Zhang, Y. I-TASSER server for protein 3D structure prediction. *BMC bioinf* **9**, 40 (2008).

7 Tamura, K., Stecher, G., Peterson, D., Filipski, A. & Kumar, S. MEGA6: molecular evolutionary genetics analysis version 6.0. *Mol. Biol. Evol.* **30**, 2725-2729 (2013).

8 Saitou, N. & Nei, M. The neighbor-joining method: a new method for reconstructing phylogenetic trees. *Mol. Biol. Evol.* **4**, 406-425 (1987).
