# Supplementary material for: Genome mining of cyclodipeptide synthases unravels unusual tRNA-dependent diketopiperazine-terpene biosynthetic machinery
Source: Nat Commun. 2018 Oct 5;9:4091. doi: 10.1038/s41467-018-06411-x (PMC6173783; doi:10.1038/s41467-018-06411-x)
Supplement: Supplementary file 3 — Descriptions of Additional Supplementary Files [file 41467_2018_6411_MOESM3_ESM.pdf]

Descriptions of Additional Supplementary Files:

File Name: Supplementary Data 1
Description: Bacteria and plasmids used in this study.

File Name: Supplementary Data 2
Description: The amino acid residues of selected CDPSs constituting the two binding pockets.
